# Unveiling the Structure–Property Relationship of MgO-Supported Ni Ammonia Decomposition Catalysts from Bulk to Atomic Structure by In Situ/Operando Studies

**DOI:** 10.1021/acscatal.3c05629

**Published:** 2024-02-08

**Authors:** Tolga H. Ulucan, Jihao Wang, Ezgi Onur, Shilong Chen, Malte Behrens, Claudia Weidenthaler

**Affiliations:** † Max-Planck-Institut für Kohlenforschung, Kaiser-Wilhelm-Platz 1, DE-45470 Mülheim an der Ruhr, Germany; ‡ Institute for Inorganic Chemistry Christian-Albrechts-Universität zu Kiel Max-Eyth-Str. 2, 24118 Kiel, Germany

**Keywords:** ammonia decomposition, in situ XRD, XAFS, operando total scattering, Ni catalysts on MgO support

## Abstract

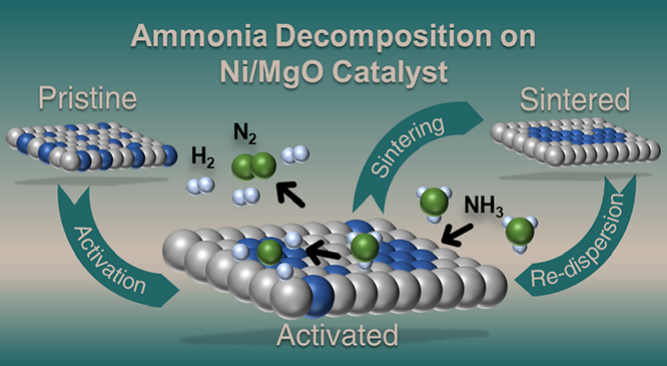

Ammonia is currently being studied intensively as a hydrogen carrier in the context of the energy transition. The endothermic decomposition reaction requires the use of suitable catalysts. In this study, transition metal Ni on MgO as a support is investigated with respect to its catalytic properties. The synthesis method and the type of activation process contribute significantly to the catalytic properties. Both methods, coprecipitation (CP) and wet impregnation (WI), lead to the formation of Mg_1–*x*_Ni_*x*_O solid solutions as catalyst precursors. X-ray absorption studies reveal that CP leads to a more homogeneous distribution of Ni^2+^ cations in the solid solution, which is advantageous for a homogeneous distribution of active Ni catalysts on the MgO support. Activation in hydrogen at 900 °C reduces nickel, which migrates to the support surface and forms metal nanoparticles between 6 nm (CP) and 9 nm (WI), as shown by ex situ STEM. Due to the homogeneously distributed Ni^2+^ cations in the solid solution structure, CP samples are more difficult to activate and require harsher conditions to reduce the Ni. The combination of in situ X-ray diffraction (XRD) and operando total scattering experiments allows a structure–property investigation of the bulk down to the atomic level during the catalytic reaction. Activation in H_2_ at 900 °C for 2 h leads to the formation of large Ni particles (20–30 nm) for the samples synthesized by the WI method, whereas Ni stays significantly smaller for the CP samples (10–20 nm). Sintering has a negative influence on the catalytic conversion of the WI samples, which is significantly lower compared to the conversion observed for the CP samples. Interestingly, metallic Ni redisperses during cooling and becomes invisible for conventional XRD but can still be detected by total scattering methods. The conditions of activation in NH_3_ at 650 °C are not suitable to form enough reduced Ni nanoparticles from the solid solution and are, therefore, not a suitable activation procedure. The activity steadily increases in the samples activated at 650 °C in NH_3_ (Group 1) compared to the samples activated at 650 °C in H_2_ and then reaches the best activity in the samples activated at 900 °C in H_2_. Only the combination of complementary in situ and ex situ characterization methods provides enough information to identify important structure–property relationships among these promising ammonia decomposition catalysts.

## Introduction

Efforts to replace fossil fuels and implement renewable hydrogen as an energy carrier have increased in recent decades. Beyond efficient production of renewable hydrogen via water electrolysis, storage and transportation of hydrogen in an economically feasible manner are the two main obstacles to a hydrogen-based energy economy.^1^ Currently, hydrogen is stored physically in pressurized gas tanks (up to 700 bar) or liquefied under cryogenic conditions (−252.9 °C).^2,3^ Furthermore, hydrogen atoms can easily diffuse through the container material due to their small size, resulting in “hydrogen embrittlement”.^4^ These methods of hydrogen storage lead to significant energy losses and reduce the overall efficiency.^5^ Another alternative is the chemical storage of hydrogen in solids such as complex metal hydrides,^6^ imides/amides,^7,8^ liquid organic hydrogen carriers (LOHC), and other small molecules.^9^ Ammonia (NH_3_) has recently gained a lot of attention as a hydrogen carrier because of its high hydrogen content (17.8 wt %) and energy density (13.6 GJ m^–3^) as well as its facilitated transportation properties since NH_3_ can be liquefied at 8 bar at room temperature. Furthermore, the global production capacity and worldwide transportation network for NH_3_ make it the most economical candidate.^10,11^

The ammonia decomposition reaction is inherently endothermic and requires high temperatures to produce high-purity hydrogen, which is essential for proton exchange membrane fuel cells (PEMFCs), as even 1 ppm of NH_3_ in H_2_ has been reported to increase the cell resistance over time.^12^ Therefore, the development of catalysts capable of lowering reaction temperatures while increasing efficiency is critical. From a thermodynamic point of view, the equilibrium conversion of NH_3_ can reach 99% at a temperature of 400 °C and 1 atm, but the reaction kinetics is sluggish.^13^ There are numerous studies in the literature reporting that ruthenium (Ru) has excellent properties and is the most active metal for the decomposition of ammonia.^14−20^ Ru on carbon nanotubes promoted by potassium has been reported to reach the thermodynamic equilibrium at temperatures as low as 450 °C.^21,22^ However, the large-scale application of these catalysts is very limited due to the cost and scarcity of noble metals. In addition, the long-term stability of both the catalyst and carbon support is a critical issue.^23^ Thus, different transition metals were considered as more abundant alternatives. Depending on the type of support, NH_3_ flow rate, or catalyst concentration, different orders of activity were found. Considering Ru/Al_2_O_3_ as the benchmark with the highest conversion at low temperatures, the activity decreases with the type of active metal in the following order: Ru > Ni > Rh > Co > Ir > Fe, Pt > Cr > Pd > Cu, Te, Se, Pb.^24^ For carbon supports, the conversion decreases with Ru > Rh ≃ Ni > Pt ≃ Pd > Fe.^21^ Over the past decade, other catalysts such as metal amides and/or imides,^25^ bimetallic catalysts,^26^ and recently also high entropy alloys^27^ have been investigated. Combinations of different active elements with different supports (Al_2_O_3_, carbon nanotubes, CeO_2_, SiO_2,_ perovskites, and MgO) and different promoters have shown improved catalytic activities.^14,28,29^ Commercial ammonia crackers for galvanization processes use nickel on alumina as catalysts but the reaction temperatures between 850 and 950 °C are very high.^14^

The Ni on MgO system was investigated in terms of the reaction kinetics. Nakamura and Fujitani reported that the NH_3_ decomposition reaction is controlled by the Ni–N binding energy^30^ which was then supported by Takahashi and Fujitani who showed that the NH_3_ dehydrogenation step is the rate-determining step.^31^ Later, Ni/La-MgO, Fe/La-MgO, and Co/La-MgO catalysts were reported to show the promoting effects of La.^32^ However, these studies mostly rely on post-mortem ex situ X-ray powder diffraction (XRPD) and X-ray photoelectron spectroscopy (XPS) studies. Such ex situ measurements are usually performed under conditions that do not represent the state of the catalysts under reaction conditions. Therefore, the interpretation of such data may be misleading. Weidenthaler et al. reported the formation of cobalt aluminate species under reaction conditions that hinder the catalytic activity by a series of in situ XRD and XPS studies on Co/γ-Al_2_O_3_ catalysts, highlighting the importance of in situ studies in revealing the true structure–property relationships.^33^

In this work, the structure–property relationship of Ni catalysts supported on MgO for ammonia decomposition is discussed. The catalysts were synthesized by two different routes, wet impregnation (WI) and coprecipitation (CP), leading to different microstructures and properties such as reducibility to obtain the active species for ammonia decomposition. A systematic and comprehensive series of ex situ XRPD, X-ray absorption fine structure (XAFS), temperature-programmed reduction (TPR), scanning transmission electron microscopy (STEM), and in situ XRPD, XPS, and operando total X-ray scattering with subsequent atomic pair distribution function (PDF) analyses experiments were performed to determine the relationship between the metal and the support structure and its effects on catalytic activity.

## Experimental Section

### Synthesis

In this study, two different synthesis routes, CP and WI, were used to study the effects of bulk synthesis and surface modification of Ni on the MgO support. Two different metal loadings, 10 atom % Ni/MgO and 20 atom % Ni/MgO indicating the ratio of Ni to Ni + Mg atoms in the catalysts, were analyzed. These molar metal loadings refer to nominal mass loadings of 13.4 and 24.9 wt % Ni/MgO, respectively. All samples were calcined at 600 °C for 2 h.

#### WI

Commercial MgO powder (Sigma-Aldrich, ≥ 99%) was impregnated with a 0.1 mol L^–1^ Ni(NO_3_)_2_ solution. The 0.1 mol L^–1^ Ni(NO_3_)_2_ solution was prepared by mixing Ni(NO_3_)_2_·6H_2_O (Alfa Aesar, 98%) with deionized H_2_O and stirring until complete dissolution. The solution was gradually added to the MgO powder, and loadings were controlled by the volume of solution added. Stirring was continued overnight to ensure homogeneous mixing without any filtration. The samples were dried at 100 °C overnight and calcined at 600 °C. Depending on the metal concentration the samples are labeled 10% Ni/MgO WI and 20% Ni/MgO WI.

#### CP

A hydroxide precursor was synthesized via a precisely controlled CP method in an automatic workstation (OptiMax 1001, Mettler Toledo). A 1 mol L^–1^ metal salt solution containing Ni(NO_3_)_2_·6H_2_O (99.9%, abcr GmbH, Germany) and Mg(NO_3_)_2_·6H_2_O (99%, Grüssing GmbH, Germany) in the above-mentioned ratios was continuously dosed with a rate of 2 g min^–1^ for 60 min into the reactor prefilled with 200 mL of distilled water. The pH value of the suspension was probed by an electrode inserted into the reactor and kept at 10.5 by computer-controlled on-demand dosing of 1.2 mol L^–1^ of NaOH solution (99%, Grüssing GmbH, Germany). At the end of the CP period, the suspension was aged in the reactor at 50 °C for 2 h. The aged products were then filtered and washed with distilled water until the filtrate showed an electrical conductivity of less than 100 μs cm^–1^. The washed product was dried at 80 °C overnight and then calcined at 600 °C for 2 h. Depending on the metal concentration, the samples were labeled 10% Ni/MgO CP and 20% Ni/MgO CP. The synthesis procedures and sample codes are summarized in Table 1.

**Table 1:** Sample Preparation Method, Calcination Conditions, and Sample Codes

synthesis	calcination	sample code
wet impregnation (WI)	600 °C/air, 2h	10% Ni/MgO WI
wet impregnation (WI)	600 °C/air, 2h	20% Ni/MgO WI
coprecipitation (CP)	600 °C/air, 2h	10% Ni/MgO CP
coprecipitation (CP)	600 °C/air, 2h	20% Ni/MgO CP

**Scheme 1:**
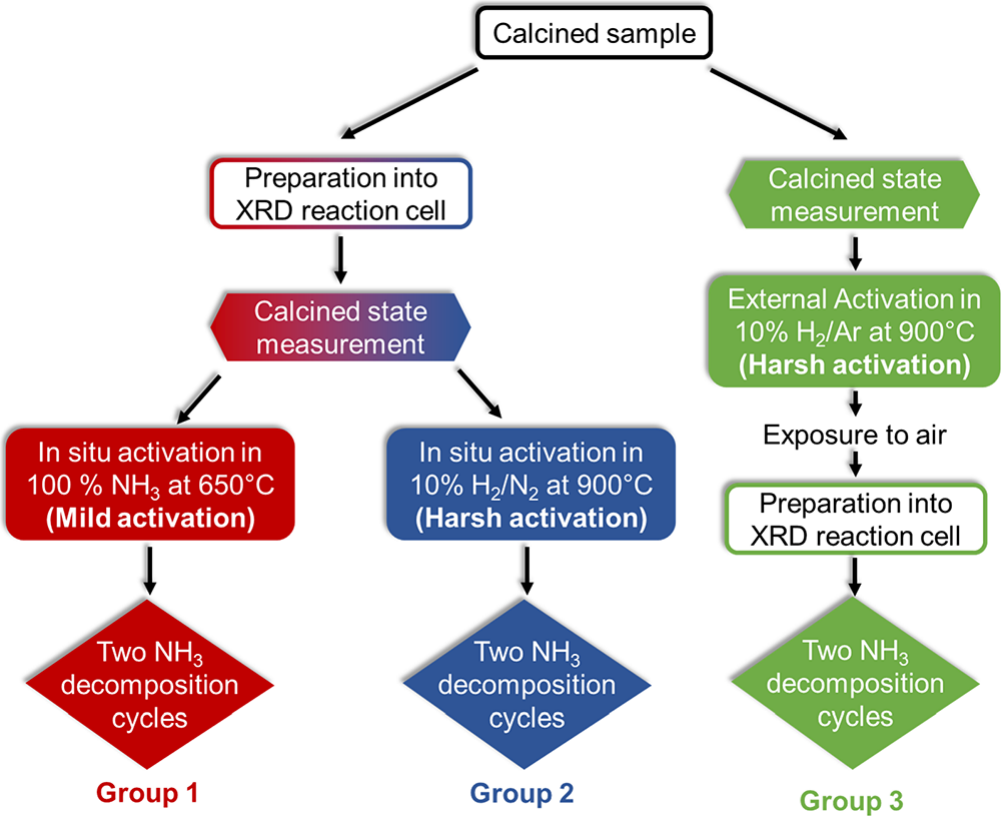
Summary of the Different Sample Treatment Procedures

To study the influence of the activation procedure of the active catalyst, two different activation routes were followed: (i) mild activation: the catalysts were exposed to 100% NH_3_ stream (flow rate: 5 mL min^–1^) in the reactor and heated from 350 to 650 °C (50 °C per step, 45 min for each step, and afterward cooling to RT). (ii) Harsh activation: the catalysts were exposed to a 10% H_2_/Ar flow (flow rate: 50 mL min^–1^) and heated to 900 °C in an external reduction unit and then cooled to RT. After activation, the samples were removed from the setup and exposed to air. After both activation procedures, catalytic experiments were evaluated in 100% NH_3_ at RT and run in repeated cycles up to 650 °C. Additionally, in situ X-ray diffraction experiments were performed under harsh activation conditions before switching to an ammonia atmosphere. The activation procedures are summarized in Scheme 1 in the section on in situ diffraction experiments.

### X-ray Powder Diffraction

Ex situ XRPD data collection was performed with a Rigaku SmartLab diffractometer equipped with a rotating anode (9 kW, 45 kV, 200 mA) in Bragg–Brentano geometry using Cu Kα_1/2_ (λ = 1.5406 Å) radiation (Cu Kβ radiation was eliminated by a nickel filter). Sufficient high resolution and counting statistics were achieved with an elliptical multilayer mirror and a HyPix-3000 multidimensional detector in 1D mode. The samples were placed on a silicon background-free sample holder, and data were collected continuously in the range of 20–90° 2θ. The data were refined using the Rietveld program TOPAS V5.^34^ Crystallite sizes expressed as column length distributions were obtained by whole powder pattern modeling (WMMP) implemented in TOPAS V6.

The volume-averaged size (*L*_Vol_) of the MgO crystallites was determined by evaluating the broadening of the diffraction peaks before ammonia decomposition and after the final cycle. The volume-averaged sizes were determined by structure-independent fitting of the profiles using the program package WinXPOW SIZE (Version 2.02, July 2003, STOE and Cie GmbH) by deconvoluting the Lorentzian and Gaussian contributions to the profile. Instrumental broadening effects were accounted for by analyzing a size standard (Si NIST 664b). These values correspond to what is commonly referred to as Scherrer crystallite size. However, the evaluation of crystallite sizes using the Scherrer approach does not account for a broad crystallite size distribution or anisotropic peak broadening and is therefore subject to large errors.

In situ XRD measurements were performed with an Anton Paar XRK900 reaction chamber mounted on a Rigaku SmartLab diffractometer. The reactor was equipped with a ceramic MACOR© sample holder with a sieve plate at the bottom to allow gas flow through the entire sample volume, simulating a plug flow reactor. The reaction chamber was connected to a gas supply system, and the gas flow parameters could be set individually for each sample, allowing the mild and harsh activation conditions mentioned above to be applied before the catalytic ammonia decomposition was started. In addition, one group of samples was activated externally, and then, in situ measurements were performed during ammonia decomposition. During catalysis, the samples were directly heated in a continuous flow of pure NH_3_ (100%, dry) (weight hourly space velocity, WHSV, 15,000 cm^3^·g_Ni_^–1^h^–1^). All samples were heated from room temperature to 350 °C at a heating rate of 20 K min^–1^ and further in steps of 50–650 °C. After reaching different temperatures, data acquisition was started (30 min per scan). To investigate cycle stability, cycling experiments were performed by heating and cooling under a continuous flow of ammonia for two cycles.

### X-ray Absorption Fine Structure

Ex situ XAFS data for calcined samples were collected at beamline P65 (PETRA III DESY, Hamburg Germany) at the Ni K edge (8333 eV) in transmission mode. Simultaneously, a Ni foil was measured as a reference. A double crystal fixed exit monochromator and ionization chambers were optimized for the Ni K-edge. To obtain a good edge jump, the catalyst powders were first ground and sieved with 20 μm mesh and then mixed with BN powder to obtain self-standing pellets. Five to eight scans were averaged to produce the final data.

### Inductively Coupled Plasma Optical Emission Spectroscopy Measurements

The inductively coupled plasma optical emission spectroscopy (ICP-OES) measurements were performed with a Spectrogreen FMX 46 spectrometer equipped with UVPlus optics analyzing the wavelength range of 165–770 nm. Solutions were prepared with aqua regia and fed through a cyclon nebulizer at a 2 mL min^–1^ rate.

### Scanning Transmission Electron Microscopy

The scanning transmission electron microscopy (STEM) measurements were performed on a Tecnai F30 G^2^ STwin instrument from Thermo Fisher Scientific equipped with a field emission gun (FEG) as the cathode and an energy-dispersive X-ray (EDX) detector from EDAX company. The acceleration voltage used in the measurement was 300 kV, and the spherical aberration coefficient was 1.2 mm. To ensure a sufficiently accurate evaluation of particle size and dispersion, over 400 particles were counted twice for each sample. The average particle size was calculated as follows (eq 1):
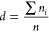
1

An estimation of the dispersion is calculated using the following equation (eq 2):
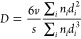
2with *d* as the average particle size, *D* as the dispersion, *v* as the volume of the Ni atom, *s* as the area one Ni atom occupies on the surface, and *n*_*i*_ as the number of particles with a diameter *d*_*i*_.^35^

### Nitrogen Adsorption

The nitrogen physisorption was conducted on a BELSORP-max apparatus (BEL JAPAN INC.) at −196 °C. Before the physisorption test, all the samples were pretreated in a vacuum at 100 °C for 2 h as an activation step. The Brunauer–Emmett–Teller (BET) surface area was obtained by evaluating the isotherm data from the *P*/*P*_0_ range of 0.05–0.3. The Barrett–Joyner–Halenda (BJH) model was used for the desorption branch of the isotherm to calculate the pore size distribution.

### Temperature-Programmed Reduction

H_2_-temperature-programmed reduction (H_2_-TPR) was performed in a BELCAT II catalyst analyzer from Microtrac MRB using a thermal conductivity detector. The calcined samples were placed into a fixed-bed microreactor and pretreated at 120 °C for 60 min with Ar (99.999%, Air Liquide). After cooling to 40 °C, H_2_-TPR was carried out in 10% H_2_/Ar at a flow rate of 80 mL min^–1^ and the catalysts were heated to 900 °C at 10 K min^–1^. For 10 and 20% Ni/MgO WI samples, the catalyst amounts used were 65 mg. The amount of the used catalyst for 10 and 20% Ni/MgO CP was 300 and 150 mg, respectively.

### Operando Ammonia Decomposition Total Scattering Experiments

Operando total scattering experiments were performed at beamline P02.1 (PETRA III DESY, Hamburg, Germany). The data were collected with a VAREX XRD 4343CT (150*150 μm^2^ pixel size, 2880*2880 pixel area) detector, placed at a distance of 300 mm from the sample with a wavelength of 0.20735 Å (energy = 60 keV) (*Q*_min_ = 0.6 Å^–1^ and *Q*_max_ = 22 Å^–1^ for the generation of the PDFs; *r*_fit_ = 0.5–20 Å, *Q*_damp_ = 0.0273 Å^–1^, and *Q*_broad_ = 0.0055 Å^–1^ determined by refinement of the PDF obtained from a Si standard). Data collection per frame was 600 s (10 frames merged, 60 s/frame). Data integration was performed with the DAWN software.^36^ PDFs were generated via the xPDFsuite software from integrated scattering data,^37,38^ and the program PDFgui was used for PDF refinements.^39^

The catalyst powders were first pelletized, ground, and meshed to obtain particle sizes between 250 and 400 μm. The samples were filled into a quartz glass flow cell with an inner diameter of 0.9 mm and a wall thickness of 0.15 mm. The powder in the capillary was fixed with quartz wool to hold the sample in place during heating under a gas flow, simulating a plug flow cell. The capillaries were then placed in a reaction cell connected to a mass flow controller (MFC) to provide pure NH_3_ (99.98%) at a weight hourly space velocity (WHSV) of 15,000 cm^3^ g_Ni_^–1^ h^–1^. The capillaries were heated by a hot air blower with a heating ramp of 10 °C min^–1^. Reaction gases were simultaneously analyzed by mass spectrometry (MS), and the pressure between the MFCs and capillaries was monitored throughout the experiment to ensure an unobstructed gas flow.

The operando experiments were performed with two different sets of samples. (i) Samples activated under harsh conditions: these samples were activated before the experiment in a separate TPR experiment under a gas flow of 10% H_2_ – 90% Ar while heating to 900 °C at a heating rate of 10 °C min^–1^. After activation, the samples were cooled to room temperature. Exposure to air was not avoided. Initial data were collected at room temperature. Then, the samples were heated in a continuous NH_3_ stream from room temperature to 400 °C at a heating rate of 10 °C min^–1^. Between 400 and 600 °C, data sets were collected every 50 °C under reaction conditions. A final data set was collected after cooling to room temperature. (ii) Calcined samples: an initial data set was collected at room temperature, and then, the sample was activated in a continuous stream of 100% NH_3_ by heating to 650 °C at a heating rate of 10 °C min^–1^. After cooling to room temperature, data were collected every 50 °C in a second cycle in the temperature range between 400 and 600 °C. A final data set was collected after the samples were cooled to room temperature.

### X-ray Photoelectron Spectroscopy

XPS spectra were collected using a SPECS GmbH instrument equipped with a PHOIBOS 150 1D-DLD hemispherical energy analyzer. The monochromatic Al Kα X-ray source (*E* = 1486.6 eV) was operated at 15 kV and 200 W. For the narrow scans, a 20 eV pass energy was applied. The medium area mode was used as the lens mode. The base pressure during the experiment in the analysis chamber was 5 × 10^–10^ mbar. All spectra were referenced to C 1s at 284.5 eV to account for charging effects.

Quasi-in situ XPS reduction experiments were performed in a reaction chamber directly attached to the XPS instrument. Since the reaction chamber cannot be operated to 900 °C, we decided to limit the temperatures to the maximum temperatures used for the catalytic tests. For all samples, XPS spectra were recorded before reduction. After the measurement, the sample was transferred from the analysis chamber into the reaction chamber, and the volume was purged with N_2_ for 1 h. After 1 h, the reaction gas was changed from pure N_2_ to 10% H_2_ in N_2_ (60 mL min^–1^). The sample was heated to the target temperature of 650 °C at a heating rate of 5 K min^–1^ and kept under a reductive atmosphere for 2 h. After reduction, the samples were cooled to room temperature in a continuous stream of 10% H_2_/N_2_ for 1 h. After cooling, the samples were transferred directly into the analysis chamber and XPS spectra were recorded.

### Catalytic Tests

The catalytic experiments were performed in a fixed-bed reactor fed with pure NH_3_ (99.98%, WHSV, 15,000 cm^3^ g_Ni_^–1^ h^–1^). The inner diameter of the reactor was 6 mm, and the catalyst powder loading was 60 mg. For the temperature-dependent conversion curves, the temperature was increased from 350 to 650 °C in steps of 50 °C. At each analysis temperature, eight measurements were recorded within 40 min under steady-state conditions using a micro-gas chromatograph (GC, 3000 Micro GC” from Inficon). The GC is equipped with two channels and TCDs. Channel A uses Ar as a carrier gas and is suitable for the detection of N_2_, O_2_, and H_2_. It is equipped with a backflush inlet with a molecular sieve column and a PLOT U precolumn. Channel B uses H_2_ as the carrier gas and is suitable for the detection of NH_3_. It is equipped with a variable inlet with a PLOTU column. Inlet and injector temperatures were set to 100 °C, and the column was set to 120 °C. A single run took approximately 2 min. After the catalytic experiment, the samples were cooled to room temperature under ammonia.

## Results and Discussion

### Precatalysts

#### Texture Properties and Compositions

The isotherms from the N_2_ physisorption experiments are shown in Figure S1. All four catalysts measured after calcination show a similar isotherm shape between type II and type IV, indicating a mixture of micro- and mesopores. The pore size distribution calculated by the BJH method also confirms a broad pore size distribution (see insets). The surface area and pore information on all catalysts are listed in Table 2. The WI samples 10 and 20% Ni/MgO WI have surface areas of 51 and 29 m^2^g^–1^. These values are lower than those of the CP samples 10 and 20% Ni/MgO CP with surface areas of 68 and 83 m^2^g^–1^. Besides, the CP samples have a higher average pore volume compared with the WI samples. The compositions were determined by ICP-OES and found to be in reasonable agreement with the nominal values in terms of Ni:Mg ratios.

**Table 2:** Textural Properties and Chemical Composition of the 10 and 20% Ni/MgO WI and CP Catalysts Determined after Calcination

sample	*S*_area_^a^ (m^2^ g^–1^)	*V*_p_^b^ (cm^3^ g^–1^)	*d*_p_^b^ (nm)	composition^c^
10% Ni/MgO WI	50.7	0.70	45.6	Ni_10_Mg_90_O
20% Ni/MgO WI	29.3	0.56	60.3	Ni_20_Mg_80_O
10% Ni/MgO CP	68.4	0.87	51.0	Ni_10_Mg_90_O
20% Ni/MgO CP	82.5	1.00	45.1	Ni_19_Mg_81_O

a Surface area calculated by the BET method.

b Pore volume and average pore size calculated by the BJH method.

c Determined by ICP-OES.

#### Temperature-Programmed Reduction

**Figure 1:**
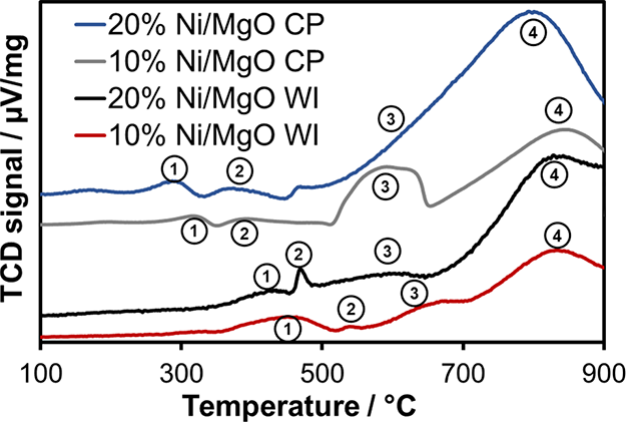
H_2_-TPR profiles of 10% Ni/MgO WI, 20% Ni/MgO WI, 10% Ni/MgO CP, and 20% Ni/MgO CP samples.

TPR data of all samples were collected together with the reduction profile of pure NiO. The comparison of NiO with the CP and WI samples (Figure S2) shows that while NiO reduces in a relatively narrow temperature range, the reduction of the catalyst samples proceeds over a very wide temperature range, as the Ni cations from the solid solution are more complex to reduce. The TCD signals measured for the catalysts are very low in intensity and should therefore not be overinterpreted. For all samples, four peaks are observed in H_2_-TPR appearing at different temperatures depending on the synthesis route and Ni loading (Figure 1). The first peaks labeled (1) are related to the reduction of nonstoichiometric Ni^3+^ to Ni^2+^ on the surface,^40,41^ which can form during calcination. The second peak (2) below 500 °C is considered to arise from the reduction of “unreacted” NiO at the surface, which is only weakly affected by the support. Bond and Sarsam assign a peak at∼ 370 °C to the reduction of free NiO.^42^ The reduction steps at higher temperatures result from the reduction of Ni^2+^, which is in strong contact with the support. In the literature, this process is described as a two-step process, with a first reduction between 590 and 630 °C of Ni^2+^ ions on or near the surface of a Mg_1–*x*_Ni_*x*_O solid solution (explained in Section 3.2) (peak 3) and a second step between 690 and 830 °C related to Ni^2+^ below the surface (peak 4).^42^ The reduction of Ni in a Mg_1–*x*_Ni_*x*_O solid solution requires a relatively high temperature because the interactions between Ni and Mg are very strong.^42^ Arena et al. also reported a correlation between the reduction temperature of Ni in the solid solution and the amount of Ni present as well as with the calcination temperature during synthesis.^43^ In the WI samples, reduction behavior is similar to that in the CP samples. The reduction peaks observed for the WI samples are shifted to higher temperatures, indicating either stronger interaction of Ni in the solid solution or larger particles.

### Activation of Catalysts

**Figure 2:**
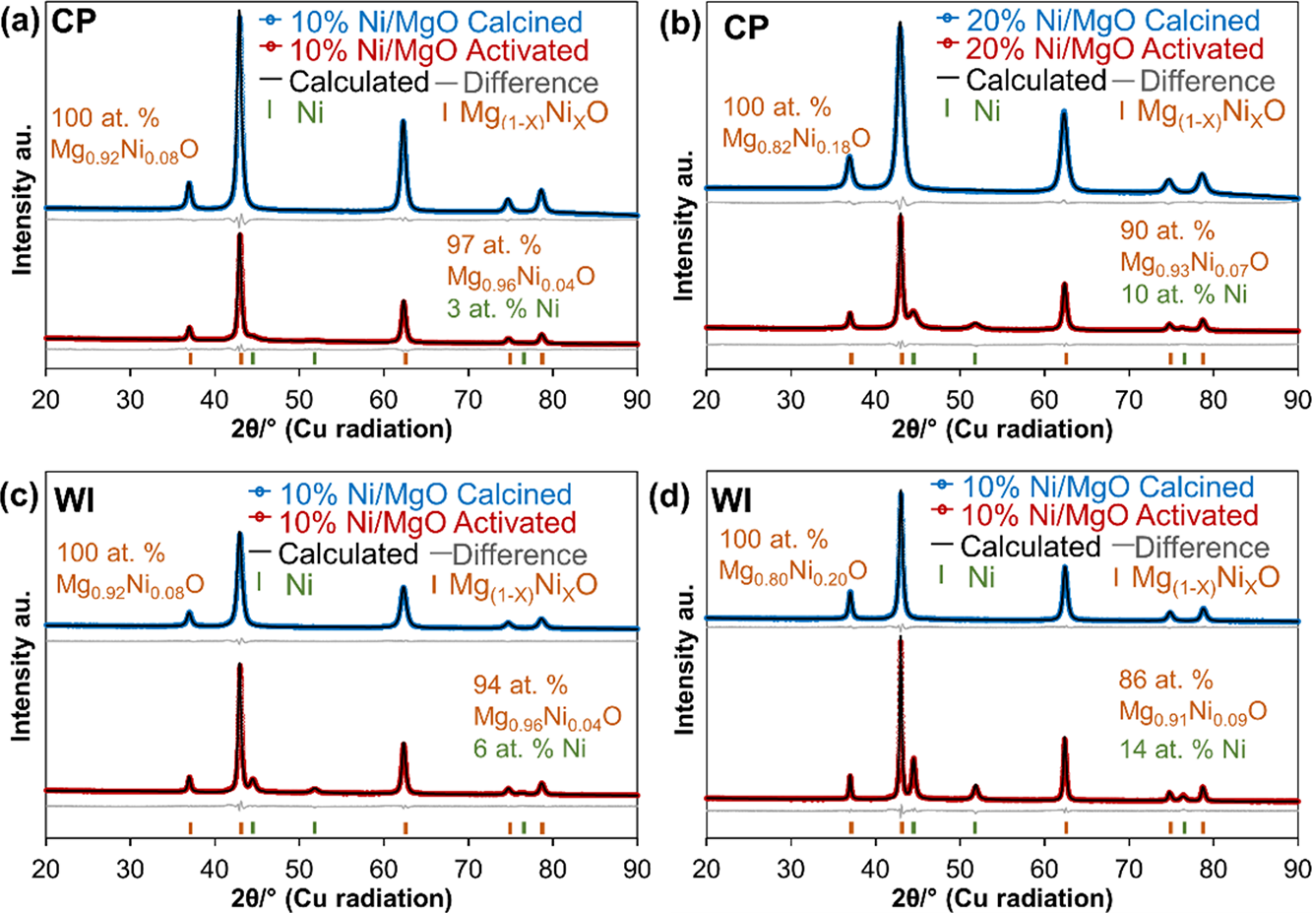
Rietveld refinement plots for the ex situ X-ray powder diffraction data. All samples either after calcination or after external activation in H_2_ have been exposed to air for the measurements. (a) 10% Ni/MgO CP calcined (top) and after harsh activation (bottom) at 900 °C in H_2_, (b) 20% Ni/MgO CP calcined (top) and after activation (bottom) at 900 °C in H_2_, (c) 10% Ni/MgO WI calcined (top) and after activation (bottom) at 900 °C in H_2_, and (d) 20% Ni/MgO WI calcined (top) and after activation (bottom) at 900 °C in H_2_. The Bragg peak positions of the Mg_1–*x*_Ni_*x*_O solid solution and metallic Ni are given as tick marks, and the phase compositions were calculated based on the Rietveld refinements.

Ex situ powder X-ray diffraction (XRPD) data were collected for all samples before and after harsh activation. In addition, the crystal structure of pure MgO obtained by CP was also analyzed (Figure S3). The lattice parameter of pure MgO was refined to 4.2170(1) Å, comparable to the literature value of 4.2198(6) Å.^44^ An averaged crystal diameter of 10 nm with a standard deviation of 5 nm was determined by WPPM. For the Ni-containing samples, structure analysis (refinement of the occupancy factor) revealed that Ni^2+^ partially replaces Mg^2+^ in the MgO structure, resulting in a Mg_1–*x*_Ni_*x*_O solid solution as already suggested by the H_2_-TPR results (Figure 2). Refinement of the data using a pure MgO phase as a structural model revealed a significant discrepancy between the observed and simulated data (Figure S4). Since the ionic radii of Mg^2+^ and Ni^2+^ in 6-fold coordination are not strongly different (0.72 Å for Mg^2+^ and 0.69 Å for Ni^2+^), a substitution has only little effect on the lattice parameter of the solid solutions (Table 3). Regardless of the Ni loading or synthesis method, no additional crystalline Ni phase is observed besides the main Mg_1–*x*_Ni_*x*_O solid solution after calcination (Figure 2). All samples activated under harsh conditions were exposed to air during the ex situ XRD experiments. The refined crystallographic parameters for all samples are summarized in Table 3. For the calcined samples, the refined occupancy parameter *x* gives a Ni content in the solid solution that is close to the values expected from the synthesis and ICP-OES results (Table 2). After harsh activation, metallic Ni forms at the expense of Ni cations in the solid solution (Figure 2). Considering the total amounts of metallic Ni, WI samples form more metallic Ni than the CP samples.

**Table 3:** Rietveld Refinement Results Obtained from the Ex Situ Diffraction Data of Calcined Samples and Samples Activated Externally under Harsh Conditions at 900 °C in H_2_

sample	lattice parameter ss^a^ / Å	lattice parameter Ni/Å	Ni in ss^a^ /atom %	reduced Ni/atom %
10% Ni/MgO CP calcined	4.2144(1)		8	
10% Ni/MgO CP harsh activation	4.2111(1)	3.5280(1)	4	3
20% Ni/MgO CP calcined	4.2138(1)		18	
20% Ni/MgO CP harsh activation	4.2100(1)	3.5270(2)	7	10
10% Ni/MgO WI calcined	4.2130(1)		8	
10% Ni/MgO WI harsh activation	4.2115(1)	3.5270(2)	4	6
20% Ni/MgO WI calcined	4.2066(1)		20	
20% Ni/MgO WI harsh activation	4.2087(1)	3.5258(1)	9	14

a ss indicates the solid solution.

#### Distribution of Ni in the Solid Solution Determined by XAFS Analysis

**Figure 3:**
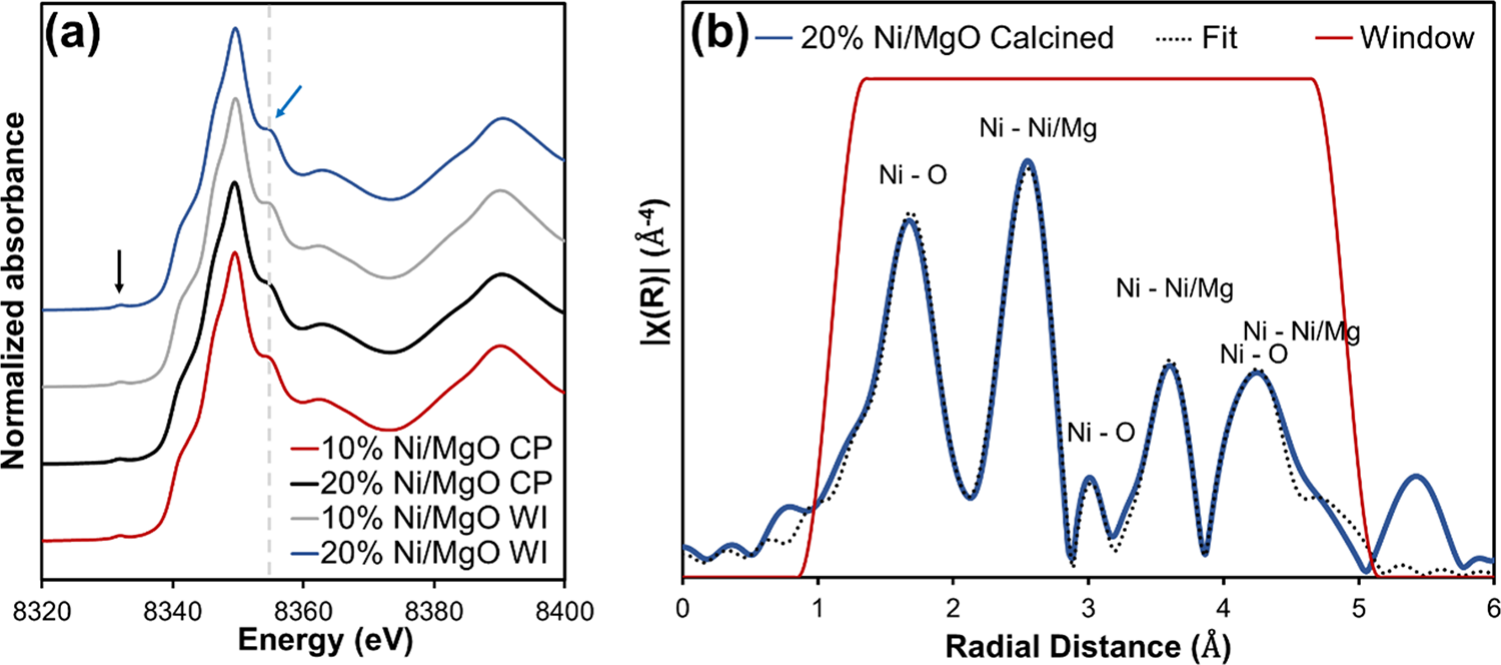
(a) Ni K-edge normalized XANES data displayed for the calcined samples. (b) Fourier transform of k^3^ weighted Ni K-edge EXAFS of 20% Ni/MgO CP calcined sample with the EXAFS fit, fitting window, and highlighted atomic interactions. Ni/Mg refers to the accumulation of both Ni–Ni and Ni–Mg interactions.

XAFS was used because of the analogous structural properties of NiO and MgO. This element-selective technique allowed for a detailed exploration of the local arrangement around the Ni atoms. Figure 3a presents the Ni K-edge XANES profiles of the calcined samples. Notably, all samples show similar features in the XANES region, which indicates the same structural environment and electronic state of the Ni atoms in the different samples. XANES spectra of Ni^2+^ within the solid solution have been examined in the literature.^45,46^ The spectra show the following characteristic features: (i) pre-edge peak around 8330 eV marked by the black arrow (left) and (ii) side peak after the main absorption peak at around 8355 eV marked by the blue arrow (right) in Figure 3a. The first feature is due to the transition from 1s to 3d, which can be used as an indicator of the symmetry around the Ni atoms. The area of this peak was reported to increase with distortion from the regular octahedron.^45^ The area of this peak is quite small and does not change between the samples, implying that oxygen atoms, forming an octahedron around the Ni atoms, are undisturbed in all calcined samples, regardless of the synthesis route or composition. Also, the second characteristic side peak, which was reported to shift to higher energies with increasing Ni content,^45^ remains stable between 10 and 20% Ni loading for both CP and WI routes as indicated by the dashed line in Figure 3a.

Figure 3b displays the Fourier transform of the k^3^-weighted Ni K-edge EXAFS of the 20% Ni/MgO CP calcined sample. Fourier transform was applied within the k range of 3–13 Å^–3^, and the fit was performed in the R range of 1.1–4.9 Å. In this range, approximately 25 independent points were identified for the fit. A sufficient signal-to-noise ratio was achieved within the Fourier transform range (Figure S5). The scattering paths used for fitting were simulated with FeFF,^47^ based on a modified structure of MgO (cubic, *Fm*3*m*, *a* = 4.21 Å) in accordance with the pair distances and lattice parameters reported by Kuzmin and Mironova.^48^ The detailed procedure can be found in Section S1. The amplitude reduction factor was determined from Ni standard measurements as 0.92. Processing and analysis of the XAFS data were performed using the Demeter software package.^49^

EXAFS fitting results for the calcined sample with 20% Ni/MgO CP shown in Figure 3b provide insights into the local structure around Ni. The first observed peak between 1 and 2 Å is attributed to the Ni–O scattering path in the first shell. This is followed by another peak between 2 and 3 Å corresponding to scattering paths with Ni–Ni and Ni–Mg atoms in the second coordination shell. Peaks occurring beyond the second shell arise from combinations of single and multiple scattering originating from Ni–Mg/Ni and Ni–O interactions. The smaller peak around 3 Å is primarily attributed to the Ni–O scattering path within the third coordination shell. Subsequent peaks are predominantly due to Ni–Ni and Ni–Mg scattering paths within the fourth and sixth shells and Ni–O scattering in the fifth coordination shell. Fourier transforms of the EXAFS data and fits for all calcined samples are shown in Figure S6.

Table 4 presents the CNs obtained by the fitting process along with the locally determined Ni/(Ni+Mg) ratios of the first six coordination shells of Ni as extracted by EXAFS analysis and the global ratios derived from ICP measurements. In the 20% Ni/MgO sample prepared via the CP route and subjected to calcination, the coordination of Ni atoms is as follows: an average of 2.1 Ni atoms and 9.9 Mg atoms in the second shell, for a total of 12 atoms; 1.4 Ni atoms and 4.6 Mg atoms in the fourth shell; and 3.6 Ni atoms and 20.4 Mg atoms in the sixth shell. Overall, the random distribution of Ni atoms in the coordination shells and the Ni/(Ni+Mg) ratio of 19 calculated from the first six shells of the EXAFS fit, which agrees with the ratio obtained by ICP, prove that a random solid solution of Mg_(1-x)_Ni_*x*_O is present throughout the sample. Detailed results of the fits for all samples can be found in Figure S6, Tables S1 and S2.

**Table 4:** Coordination Numbers Obtained from EXAFS Modeling of the Calcined Samples up to the Sixth Coordination Shell and Local Ni Compositions Obtained by EXAFS Compared to the Global Ni Compositions Obtained by ICP

		10% Ni/MgO CP	20% Ni/MgO CP	10% Ni/MgO WI	20% Ni/MgO WI
1st shell	Ni–O	6.2 ± 0.5	6.3 ± 0.3	6.3 ± 0.5	5.7 ± 0.5
2nd shell	Ni–Ni	0.9	2.1	0.6	2.3
	Ni–Mg	11.1	9.9	11.4	9.6
3rd shell	Ni–O	7.6 ± 2.1	7.6 ± 2.0	7.8 ± 2.3	8.1 ± 2.5
4th shell	Ni–Ni	1.9	1.3	1.6	0.8
	Ni–Mg	4.1	4.7	4.4	5.2
5th shell	Ni–O	24	24	24	24
6th shell	Ni–Ni	0.5	3.5	2.5	5.2
	Ni–Mg	23.5	20.5	21.5	18.8
local Ni/(Ni+Mg)% by EXAFS		10 ± 1.8	19 ± 0.7	13 ± 1.1	22 ± 0.7
global Ni/(Ni+Mg)% by ICP		10	19	10	20

Any inhomogeneity in the system, such as a Ni-rich surface or Mg-depleted regions in the bulk, would lead to discrepancies between the global chemical composition determined by ICP and the local composition obtained by EXAFS. For CP samples, local and global Ni/(Ni+Mg) ratios match perfectly. However, for WI samples, the local Ni/(Ni+Mg) ratio is higher than the global chemical composition determined by ICP. This discrepancy indicates that the Ni atoms in the WI samples are not as homogeneously distributed as in the CP samples, resulting in local Ni-rich and Ni-depleted regions.

#### Distribution of Metallic Ni Nanoparticles after Activation Investigated by Ex Situ STEM

**Figure 4:**
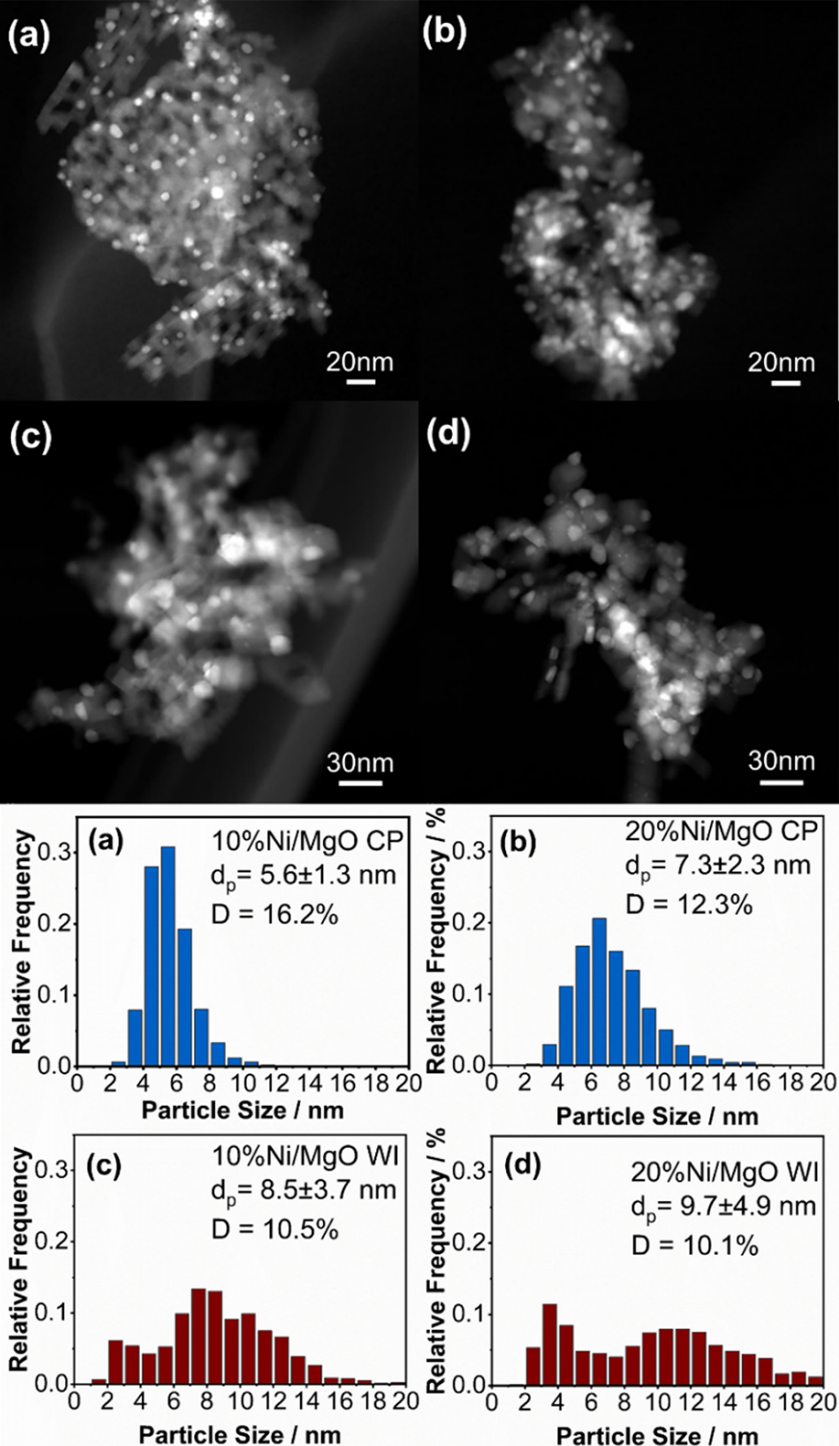
Representative STEM images of 10% (a), 20% (b) Ni/MgO CP and 10% (c), 20% (d) Ni/MgO WI activated under harsh conditions with corresponding particle size distribution and dispersion.

The ex situ STEM images in Figure 4 show the distribution of metallic Ni particles on the MgO support after activation under harsh conditions. For both WI and CP catalysts, an increase in Ni loading results in larger particle sizes and lower dispersion. The average particle sizes of 5.6 and 7.3 nm for 10 and 20% Ni/MgO CP are smaller than those of the WI catalysts with average particle sizes of 8.5 and 9.7 nm. The highest dispersion of 16% is observed for 10% Ni/MgO CP, followed by 20% Ni/MgO CP with a dispersion of 12%.

The variation in particle size and distribution of metallic Ni can be attributed to distinct Ni-rich and Ni-depleted areas in the WI samples, whereas CP samples exhibit a more uniform distribution of metallic nickel as a result of the more homogeneous composition of the solid solution after calcination, as described in the XAFS section. In the WI samples, Ni-rich domains within the solid solution likely lead to larger particles of the metallic nickel phase after the activation process. In the context of WI, areas with high nickel concentration experience an earlier reduction to metallic nickel, leading to larger particles compared to those in Ni-depleted regions. This growth discrepancy results from the diffusion-controlled formation of the metallic nickel phase, which is more likely to nucleate and grow in a Ni-rich region than in a Ni-depleted region. In contrast, the nickel cations in the CP samples are uniformly distributed in the solid solution, resulting in a more uniform particle size distribution of metallic nickel particles after reduction (Figure 4).

#### Surface Analysis by X-ray Photoelectron Spectroscopy

Quasi-in situ XPS experiments were performed to study how the surface changes during activation in hydrogen. Since the experimental setup does not allow activation of the samples at 900 °C, we decided to limit the temperature to the maximum temperature used for catalysis, which was 650 °C. The Ni 2p core level XPS data collected for all samples after synthesis and calcination at 600 °C show the presence of Ni^2+^ on the surface (Figure S7). The evaluation of the spectra reveals that surface Ni exists as nickel hydroxide rather than NiO, which likely is a result of the hygroscopic nature of MgO-based solid solution in humid air. After the reduction in 10% H_2_ in N_2_ at 650 °C for 1 h and subsequent cooling to room temperature in the same gas atmosphere, all spectra show the presence of metallic nickel. However, the temperatures are not sufficiently high to reduce all of the surface nickel to the metallic state, which is in agreement with the TPR data. This additional information underlines once again that activation at high temperatures is necessary to transform enough nickel species to the active metallic state. It has to be emphasized that the sample 20% Ni/MgO WI shows the highest amount of metallic Ni already after reduction at 650 °C but this does not necessarily correlate with the catalytic activity because other factors such as the crystallite size have to be considered as well. As shown by the in situ XRD data (Figure S8), 20% Ni/MgO WI forms larger Ni crystallites compared to the other samples which is a disadvantage.

### Structural Analysis under Reaction Conditions

#### Monitoring Long-Range Correlations by In Situ Ammonia Decomposition XRPD

The in situ XRPD experiments, performed for the samples containing 10 and 20 atom % Ni after synthesis and calcination at 600 °C, can be divided into three groups (Scheme 1) as shown below:

**Group 1**: Samples were activated in the XRD reaction chamber by heating to 650 °C in 100% NH_3_ (mild activation). After the activation step, the samples were cooled to room temperature, and the NH_3_ decomposition reaction was monitored during two more cycles between room temperature and 650 °C.
**Group 2**: Samples were activated in the XRD reaction chamber at 900 °C under 10% H_2_/N_2_ flow (in situ harsh activation). After cooling the samples to room temperature, the gas stream was switched to 100% NH_3_ and NH_3_ decomposition was monitored during two cycles between room temperature and 650 °C.
**Group 3**: Activation was performed externally at 900 °C in a flow of 10% H_2_/Ar (harsh activation ex situ). The samples were cooled to room temperature and exposed to ambient conditions. Then, the samples were prepared in the XRD reaction chamber and the NH_3_ decomposition reaction was monitored for two cycles between room temperature and 650 °C.

The Scherrer method was employed to estimate the crystallite sizes of metallic Ni and MgO support at 650 °C under reaction conditions. (Section S2, Table S3). The data evaluation reveals that Ni crystallites of the WI samples are larger during the reaction at 650 °C than for the CP samples (Group 2). The temperature-dependent changes of the lattice parameters of MgO measured during the last cycle were plotted against the temperature and show the expected thermal expansion (Figure S9).

**Figure 5:**
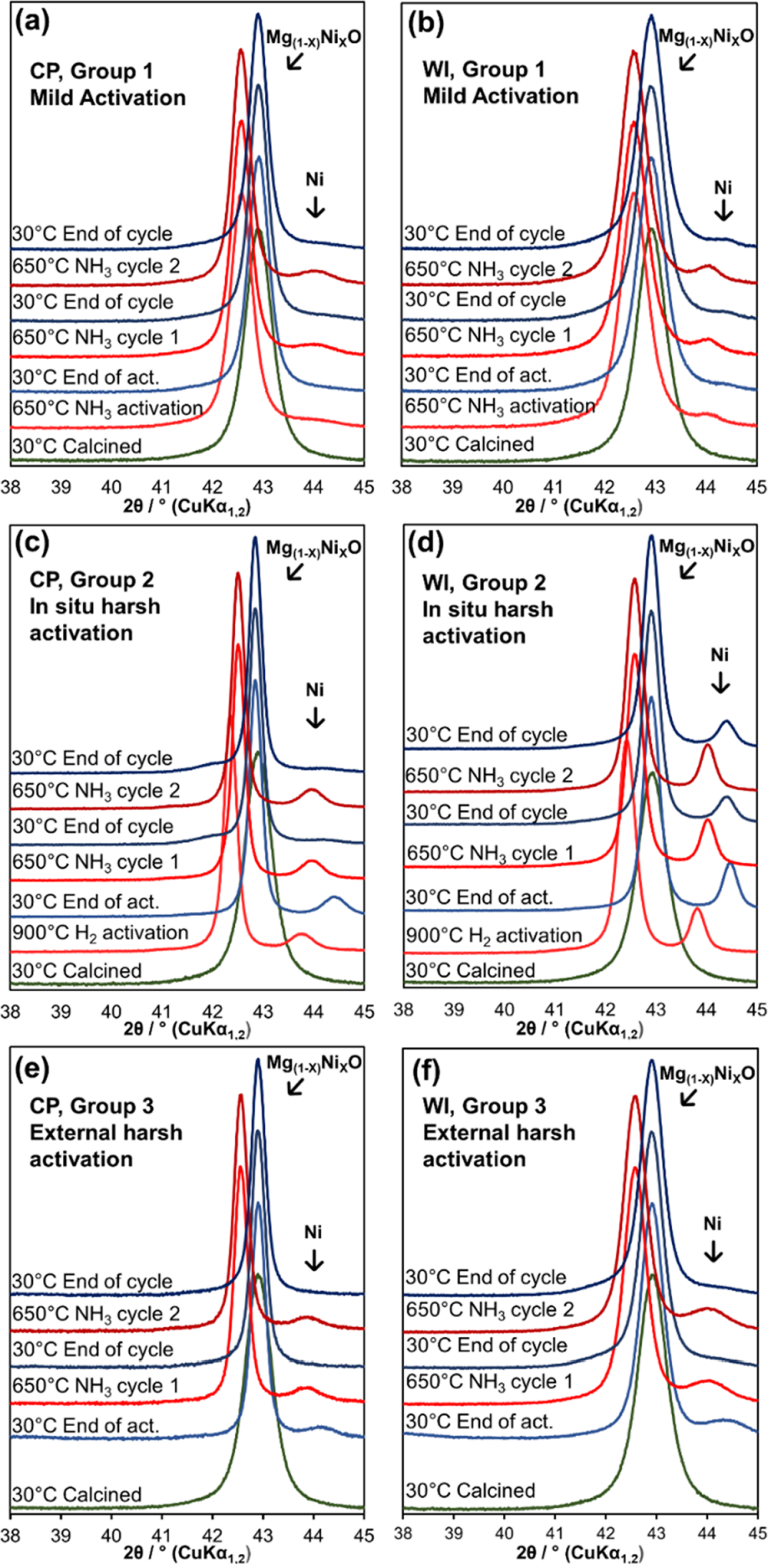
Sections of the in-house in situ XRD powder patterns obtained from samples (a) 10% Ni/MgO CP Group 1, (b) 10% Ni/MgO WI Group 1 samples activated under mild conditions. (c) 10% Ni/MgO CP Group 2 and (d) 10% Ni/MgO WI Group 2 samples activated in situ under harsh conditions. (e) 10% Ni/MgO CP Group 3 and (f) 10% Ni/MgO Group 3 WI activated externally under harsh conditions.

Figure 5 presents magnified in situ XRPD patterns collected for both 10% Ni/MgO samples in all three groups. Starting at room temperature, the XRD patterns at the bottom display the state of the catalyst after preparation and calcination. The patterns above show the states of the catalysts at high temperatures during activation and after cooling to room temperature. In addition, the patterns were obtained at 650 °C during the first and second ammonia decomposition cycles as well as after cooling after the cycles.

Figure 5a,b displays the data of 10% Ni/MgO CP and 10% Ni/MgO WI samples activated in NH_3_ at 650 °C followed by two catalytic decomposition cycles (Group 1). In both cases, the reflections assigned to metallic Ni appeared at 650 °C during activation and catalytic cycles but disappeared almost completely during cooling under a NH_3_ flow. The reflections of metallic Ni are sharper for the WI sample than those for the CP sample, indicating the formation of larger Ni crystallites for the WI samples in accordance with the ex situ STEM results. Figure 5c,d displays the XRD data of 10% Ni/MgO CP and 10% Ni/MgO WI activated in situ in a flow of 10% H_2_/N_2_ at 900 °C followed by two catalytic decomposition cycles (Group 2). Higher temperatures and the use of H_2_ gas during activation led to significantly sharper Ni reflections compared with Group 1. In contrast to Group 1, the metallic Ni reflections did not completely disappear when the samples were cooled under H_2_ flow; instead, they became slightly sharper due to the lower thermal disorder during cooling. The high-temperature measurement performed during the first ammonia decomposition cycle indicated no change in the metallic Ni phase. After cooling under NH_3_ flow, the reflections belonging to the metallic Ni phase disappeared for the CP sample, similar to Group 1. For the WI samples, the reflections lost intensity and broadened. The second ammonia decomposition cycle is similar to the first cycle, with the metallic Ni phase reappearing at high temperatures and decreasing to some extent after cooling. In addition, a broad and low-intensity reflection forms at around 41° 2θ, which cannot be assigned unambiguously (Figures S10 and S11). Initially, it was suspected that it belonged to nickel nitrides such as Ni_3_N or Ni_4_N. However, a comparison of the simulated powder patterns based on the crystallographic data of both phases and the incorporation of the structures into the Rietveld refinement did not explain this additional reflection. The unidentified reflection appears directly after harsh activation in hydrogen but also after mild activation during cycling ammonia decomposition, and it persists during further cycles. In addition, ex situ XPS data were collected after the catalytic tests for selected samples to probe the formation of potential surface nickel nitrides. However, neither the survey scans nor the high-resolution XP scans show the presence of N species, and thus, nitride formation on the surface is excluded (Figure S12). For this reason, the search was expanded to potential Mg–Ni intermetallics. A potential match was found for MgNi_3_, and the Rietveld refinement including the structure of MgNi_3_ shows a good match between observed and simulated data (Figures S10 and S11).

Figure 5e,f shows the data of 10% Ni/MgO CP and 10% Ni/MgO WI externally activated in a 10% H_2_/N_2_ flow at 900 °C and exposed to ambient conditions followed by two catalytic decomposition cycles (Group 3). Before starting the reaction, both samples show broad reflections belonging to metallic Ni. The reflections become slightly sharper during the ammonia decomposition cycles but disappear after cooling. In situ XRD powder patterns for 20% Ni/MgO collected under the same conditions are provided in Figure S8. In contrast to 10% Ni loading, these samples have higher amounts of metallic Ni after activation and therefore have higher amounts of the remaining metallic Ni after cycling with NH_3_.

**Figure 6:**
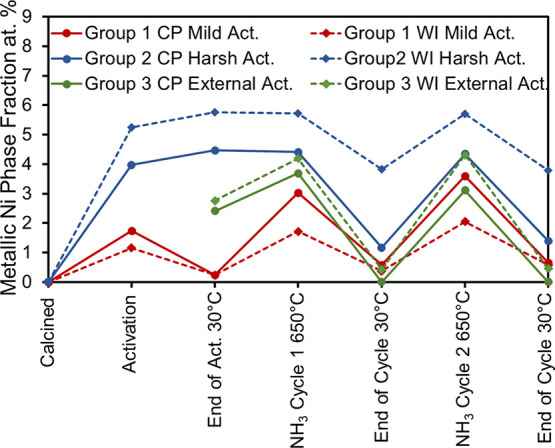
Amounts of metallic Ni formed from the solid solution during ammonia decomposition. The quantification was performed by Rietveld refinements of in situ data for 10% Ni/MgO. Results obtained for Group 1 (mild activation) are represented by red lines, results for Group 2 (harsh activation) are represented by blue lines, and the data for Group 3 (external activation) are represented by green lines. Solid lines represent CP and dashed lines represent WI samples. (Error bars are smaller than data points).

Furthermore, a comprehensive quantitative analysis using Rietveld refinements was conducted for all temperature-dependent data acquired during activation and NH_3_ decomposition. The amount of metallic Ni segregated from the solid solution for 10% Ni/MgO CP and 10% Ni/MgO WI as a function of the activation history is given in Figure 6. After calcination, all samples contain primarily a crystalline component consisting of a 100% solid solution of Mg_1–*x*_Ni_*x*_O. During activation of Group 1 samples under mild conditions, small amounts of metallic Ni (1–2 atom %) formed in both CP and WI samples. At the end of the activation, the metallic phase was reduced to below 0.5 atom % by cooling in NH_3_ The amount of the metallic Ni formed at high temperatures and remained after cooling down to 30 °C increases slightly after each cycle during mild activation.

Activation under harsh conditions leads to higher amounts of metallic Ni for Group 2 (4 atom % for CP and 6 atom % for WI). Cooling in H_2_ and heating under NH_3_ for catalytic decomposition only slightly promotes this behavior. In contrast, cooling under NH_3_ facilitates the dispersion of the formed metallic particles, leading to a decrease in the amount of metallic Ni after each cycle for both the CP and WI samples. The amount of the metal phase remaining after cooling shows remarkable differences between CP and WI samples: For CP, 72% of the reduced metallic Ni is recovered after cooling, while for WI, this recovery is much at 32%.

External activation under harsh conditions for Group 3 samples follows the same trend observed for Group 1 samples, with about 4 atom % reduced phase upon catalytic conversion at 650 °C and disappearance of the Ni reflections after cooling to 30 °C.

#### Monitoring Short- to Medium-Range Correlations by Operando X-ray Total Scattering Experiments

PDF analysis was employed to examine the behavior of Ni nanoparticles during cooling under an ammonia flow since PDF is sensitive to pair correlations below the nanometer scale, whereas XRD sensitivity is limited to a few nanometers. Two sets of treatment procedures were followed for these experiments: Group 1 and Group 3 as described in Scheme 1 and the experimental section. PDF analysis performed on the total X-ray scattering data provided insights into local atomic-scale structural changes that occur throughout the catalytic process. For this purpose, crystal structure data of MgO, NiO, and metallic Ni were included in the PDF analysis. The experimental data were refined against the model structures in the range between 0.5 and 20 Å. The refined temperature-dependent in situ PDFs are shown in Figures S13 and S14. Simultaneously, collected MS data of the outcoming gases for 10% Ni/MgO samples from Group 1 and Group 3 are listed in Figure S15. The MS signals obtained for NH_3_, N_2,_ and H_2_ ensure that data collection took place under reaction conditions. The PDF fits and corresponding phase fraction results for the samples synthesized via CP and WI methods before and after activation from Groups 1 and 3 are given in Figures S16 and S17. The phase compositions based on the PDF refinements show only a slight deviation from those based on Rietveld refinements (Figure 2).

**Figure 7:**
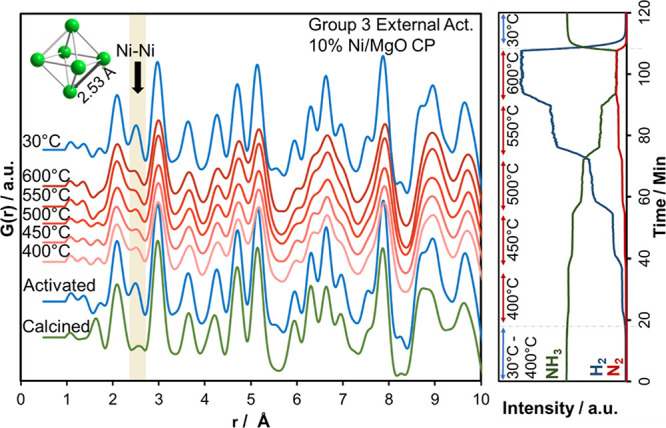
Atomic PDFs derived from the total scattering data collected for 10% Ni/MgO CP (Group 3): calcined, activated, under reaction conditions and after reaction with simultaneously collected MS data.

PDF refinements indicate that the catalysts synthesized by both CP and WI methods with 10 and 20 atom % loadings consist of MgO and NiO forming a solid solution without metallic Ni in the starting materials (Figures S16 and S17). The analysis of the calcined samples was performed to ensure that there are no short- to medium-range correlations that are not visible in XRD. Figure 7 shows operando PDF data collected from calcined, activated, during reaction and after reaction states of the 10% Ni/MgO CP sample from Group 3. In the activation treatments, the most striking indication of the changing local environment is the pair correlation that occurs at around 2.5 Å, corresponding to the Ni–Ni coordination in metallic Ni. This indicates that Ni^2+^, originally incorporated into the Mg_1–*x*_Ni_*x*_O solid solution, is partially reduced to metallic Ni by the activation treatments of the samples. This Ni–Ni pair correlation is conserved during the reaction and, unlike in the in situ XRD results, becomes even more prominent after the reaction is completed and the temperature is down to 30 °C. This discrepancy between XRD and PDF analysis indicates that reduced Ni nanoparticles get smaller and redispersed into smaller domains below the nanometer scale, which becomes invisible to XRD but can still be detected by PDF analysis. This proves that the dispersed Ni atoms in nanoparticles do not go back into solid solution.

The qualitative PDF analysis shows that the amount of metallic Ni formed after the activation does not exceed 50% of the total Ni loading during the catalytic tests and remains almost constant during the reaction (Figure S18).

### Catalytic Testing

**Figure 8:**
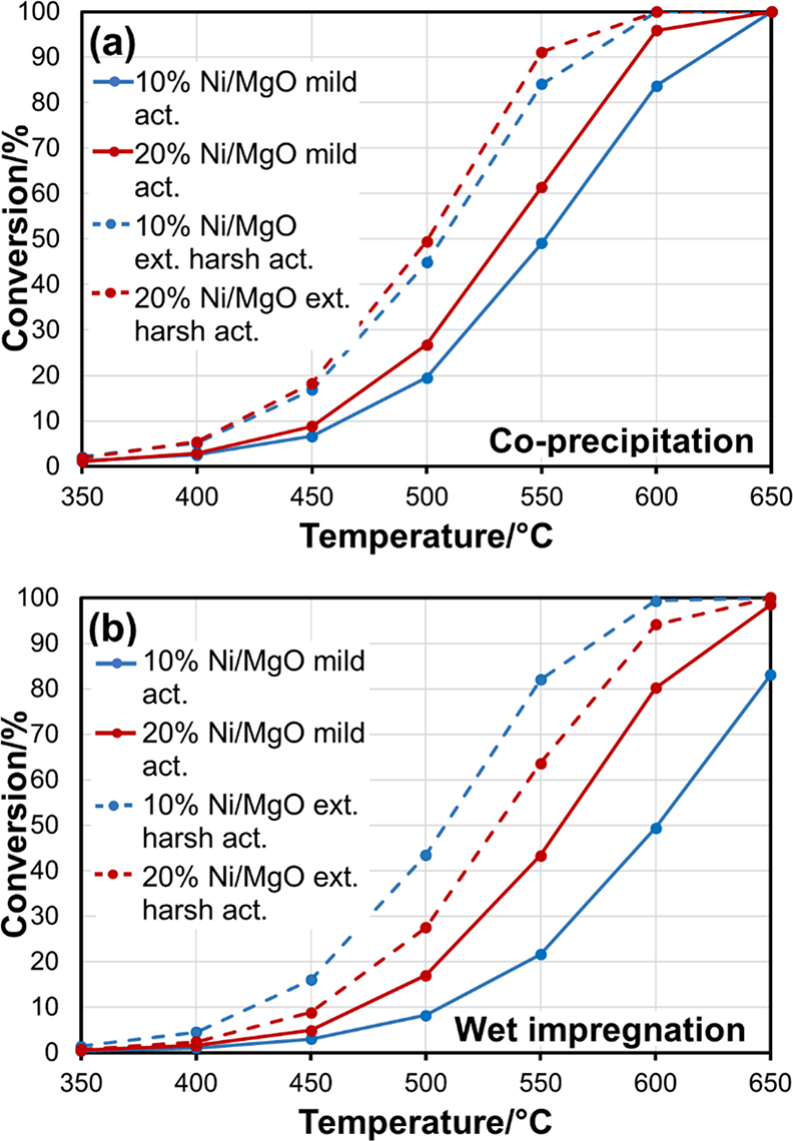
Catalytic test results of (a) CP and (b) WI samples activated under mild and harsh (externally) conditions. NH_3_ conversion curves belong to the second decomposition cycle after activation.

Catalytic tests were performed with the CP and WI samples for the two different Ni loadings, 10 and 20%. Since the catalysis reactor does not allow reaction temperatures above 800 °C, the activation was performed externally at 900 °C in H_2_ (Group 3) under harsh conditions. Figure 8 shows the conversion curves of the samples of Group 1 (mild activation in NH_3_ at 650 °C) and Group 3 (external activation under harsh conditions at 900 °C in H_2_) during the second ammonia decomposition cycle. The samples activated under harsh conditions at 900 °C (Group 3) show the highest conversions among the CP samples. At 550 °C, between 80 and 90% of NH_3_ is decomposed at a WHSV of 15,000 cm^3^ g_Ni_^–1^ h^–1^, which is comparable to literature data of conventional oxide-supported Ni catalysts measured under similar flow rates, temperatures, and Ni loadings (Table S4).^21,28,32,50^

In comparison,

- Mild activation conditions prove to be insufficient to reduce the homogeneously distributed Ni atoms, yielding NH_3_ conversions of only 50–60% at 550 °C for CP samples and even lower for WI samples. This observation is also supported by the in situ XPS experiments, which show that a significant portion of Ni remains unreduced under mild conditions up to 650 °C.
- Higher Ni loading slightly improves NH_3_ conversion under both mild and harsh activation of the CP samples without inducing sintering. This improvement is attributed to the formation of smaller and homogeneously distributed Ni particles after activation, as illustrated in Figure 4.
- For WI samples activated under mild conditions, higher Ni loading also proves to be advantageous. Conversely, lower Ni loading leads to higher NH_3_ conversions under harsh activation conditions. These results indicate that it is not necessary to work with higher Ni loadings (e.g., 20%). In fact, higher loadings lead to sintering of the reduced Ni particles, as can be seen in the article size distributions presented in Figure 4.

To investigate the effect of the activation gas alone, another set of catalyst tests was performed in which the samples were activated at 650 °C with a 10% H_2_/N_2_ stream instead of NH_3_, as in Group 1. Figure S19 shows the conversion curves obtained during the second ammonia decomposition cycle. Regardless of the Ni loading, the WI samples show 50% conversion at 530 °C, whereas the CP samples need temperatures of 510 °C to convert 50% of NH_3_. Both the CP and WI samples show higher conversion compared to mild activation under a NH_3_ flow.

The apparent activation energies (*E*_a_) and the pre-exponential factors (A) (Table 5) were extracted from the NH_3_ conversion data using the Arrhenius equation (Figure S20). Slot et al. reported that factor *A* is correlated with the frequency of collisions between reactant molecules.^51^ The number of active sites on the surface has a direct influence on *A*. Large numbers for *A* mean a higher number of active sites on the surface and a shorter time for migration between active sites and, thus, faster reactions. As expected, the most active samples 10 and 20% Ni/MgO CP (harsh activation) have the lowest activation energies. The 20% Ni-loaded sample has a slightly higher *E*_a_, but this is compensated by 1.2 times the number of active sites. On the other hand, mildly activated samples of this synthesis method have very high activation energies, which hinder the reaction rate regardless of the number of active sites. Sample 10% Ni/MgO WI has the lowest number of active sites when activated under mild conditions, suggested by a slower reaction rate. When the Ni loading increases to 20%, this number increases significantly, but *E*_a_ also reaches the maximum of all samples, limiting the successful conversion at low values. Harsh activation of the WI samples improves the overall conversion rates by decreasing *E*_a_ and increasing the number of active sites. In contrast, for the CP samples, a loading of 10% Ni results in a higher conversion than the 20% Ni loading because although the latter has the highest number of active sites, it also has the second highest *E*_a_ of all samples. Factor A and the experimentally determined dispersion values of Ni particles are consistent with each other. Higher Ni loading in both the CP and WI samples led to the formation of more densely packed Ni particles (low dispersion values), which increased the factor A factors due to a higher frequency of collisions.

**Table 5:** Activation Energies, *E*_a_, and the Pre-Exponential Factors, A, Determined from the Arrhenius Plots

sample	activation condition	*E*_a_/kJ mol^–1^	*A*/s^–1^
10% Ni/MgO CP	mild activation	91	2884
	harsh activation^a^	81	3001
20% Ni/MgO CP	mild activation	94	6219
	harsh activation^a^	84	3604
10% Ni/MgO WI	mild activation	92	1517
	harsh activation^a^	89	4576
20% Ni/MgO WI	mild activation	102	14,948
	harsh activation^a^	97	19,079

a Activated externally under harsh conditions.

To test the stability of the catalysts, the samples with the highest Ni content were tested as these samples are the most prone to sintering over time. The samples were activated externally under harsh conditions (Group 3) and subjected to two ammonia decomposition cycles. Afterward, 20% Ni/MgO CP was heated to 530 °C and 20% Ni/MgO WI to 560 °C, corresponding to 70–75% conversion for both samples. The conversion measured during 60 h shows no indication of a decrease in activity for either sample (Figure S21).

## Conclusions

In this work, Ni/MgO catalysts for ammonia decomposition with 10 and 20% Ni loading were synthesized by WI and CP methods, and the influence of different activation strategies on the catalytic activity was investigated. A combination of different complementary in situ and operando characterization techniques was applied to gain deep insights into the structure–property relationships. Crystal structure refinements revealed that the as-synthesized catalysts form Mg_1–*x*_Ni_*x*_O_2_ solid solutions after calcination. Since conventional powder diffraction is only sensitive for crystalline compounds with crystalline domains above ∼2 nm, local characterization probes such as total X-ray scattering and subsequent PDF analysis and XAS were applied. The evaluation of the XAS data revealed an inhomogeneous Ni distribution in the solid solutions obtained by WI, while CP leads to structures with a more homogeneous Ni distribution. The dispersion of metallic Ni was demonstrated by STEM, which showed smaller and more uniform Ni particles in the CP catalysts. Due to the homogeneously distributed Ni cations in the solid solution structure, CP samples are more difficult to activate and require harsher conditions to reduce Ni. The activity increases steadily from the samples activated at 650 °C in NH_3_ (Group 1) to the samples activated at 650 °C in H_2_ with the best activity in the samples activated at 900 °C in H_2_ (Group 3). Higher Ni loading also leads to slightly better activity, as the metallic Ni particles are well-distributed, and sintering does not seem to be a problem. For the CP samples, the slightly increased activation energy at higher loading is compensated by the increased number of active sites. However, for the WI samples activated under harsh conditions, higher Ni loading results in lower activity, probably due to sintering of the Ni particles. The increased Ni loading leads to a drastic increase in the activation energy that cannot be compensated by an increased number of active sites. Therefore, the WI samples do not require a high Ni loading for a sufficiently good conversion.

In situ XRD studies revealed that cooling under NH_3_ causes a decrease in crystallite size and/or redispersion of the reduced metallic Ni particles, making the catalyst undetectable by conventional XRD. The operando PDF studies confirmed that metallic Ni remains but the units are in the subnanometer range. Operando PDF analysis also showed that only about half of all Ni ions were reduced to the metallic state. This result was confirmed by operando XRD and in situ XPS data.

In conclusion, solid solutions of NiO and MgO have great potential as precursors for ammonia decomposition catalysts since the size and distribution of the active Ni phase can be controlled by the synthesis and the choice of certain activation parameters. Extensive studies under the reaction conditions have also clearly shown that high catalyst loadings are not always required. The complementary characterization methods have provided a deep insight into the structure–property relationships for this system and have shown that catalysts for ammonia decomposition are dependent on many parameters, which, in turn, are interrelated.

## Supporting Information Available

Detailed procedure of EXAFS modeling and refinement; crystallite size determination procedure and results; physisorption isotherms and pore size distribution data for all calcined samples; comparison of H_2_-TPR profiles of NiO with two samples; Rietveld refinement of 20% Ni/MgO CP with only MgO as fitting phase; XAFS spectra of all calcined samples in k-space; EXAFS data and fits for all calcined samples in r-space; XPS spectra of all samples before and after reduction; in situ XRD powder pattern sections of 20% Ni-loaded samples; lattice parameter refinements of 10% Ni-loaded samples; Rietveld refinements focusing on small reflection at around 41° 2θ; XPS spectra of CP samples with N 1s region, showing the lack of nitrides; PDF data and refinements for all samples; MS data collected during operando total scattering measurements; PDF refinements before and after activation with phase compositions; metallic Ni phase fractions obtained by PDF refinements; catalytic test results for samples activated under H_2_ without exposure to air; Arrhenius plots of the NH_3_ conversion curves; stability tests for 20% Ni-loaded samples; EXAFS fitting parameters at Ni K edge for CP samples; EXAFS fitting parameters at Ni K edge for WI samples; volume-averaged crystallite sizes determined by the Scherrer method from in situ XRD data collected during ammonia decomposition at 650 °C; and previously reported conventional oxide-supported Ni catalysts (PDF)

## ABBREVIATIONS

LOHC: liquid organic hydrogen carriers
NH_3_: ammonia
PEMFC: proton exchange membrane fuel cells
Ru: ruthenium
XRPD: X-ray powder diffraction
XPS: X-ray photoelectron spectroscopy
WI: wet impregnation
CP: coprecipitation
XAFS: X-ray absorption fine structure
TPR: temperature-programmed reduction
STEM: scanning transmission electron microscopy
PDF: pair distribution function
WMMP: whole powder pattern modeling
*L*_Vol_: volume-averaged size
ICP-OES: inductively coupled plasma optical emission spectroscopy
FEG: field emission gun
EDX: energy-dispersive X-ray
BET: Brunauer–Emmett–Teller
BJH: Barrett–Joyner–Halenda
(H_2_-TPR): H_2_-temperature-programmed reduction
MFC: mass flow controller
WHSV: weight hourly space velocity
MS: mass spectrometer
