# Supplementary material for: Unveiling the Structure–Property Relationship of MgO-Supported Ni Ammonia Decomposition Catalysts from Bulk to Atomic Structure by In Situ/Operando Studies
Source: ACS Catal. 2024 Feb 8;14(5):2828–41. doi: 10.1021/acscatal.3c05629 (PMC10913046; doi:10.1021/acscatal.3c05629)
Supplement: Supplementary file 1 — cs3c05629_si_001.pdf [file cs3c05629_si_001.pdf]

Supplementary information for

Unveiling the structure-property relationship of MgO-supported Ni ammonia decomposition catalysts from bulk to atomic structure by *in situ/operando* studies

Tolga H. Ulucan[1], Jihao Wang[2], Ezgi Onur[1], Shilong Chen[2], Malte Behrens[2], Claudia Weidenthaler[1,*]

[1]Max-Planck-Institut für Kohlenforschung, Kaiser-Wilhelm-Platz 1, DE-45470 Mülheim an der Ruhr, Germany

[2]Institute for Inorganic Chemistry Christian-Albrechts-Universität zu Kiel Max-Eyth-Str. 2, 24118 Kiel (Germany)

*Corresponding author: weidenthaler@mpi-muelheim.mpg.de

Supplementary Notes

Section S1. EXAFS modelling and refinement procedure

Two models for 10% and 20% Ni loading were constructed for the EXAFS refinements as follows: First, a standard MgO (cubic, *Fm*3*m*, a=4.21 Å) crystal with a radius of 9 Å was generated. The central Mg atom was replaced by Ni. Then Mg atoms were randomly replaced by Ni atoms according to the overall composition in each Mg coordination shell of the Ni atom. Finally, the lattice parameters and atomic distances between the Ni and Mg atoms were modified to introduce the lattice strain caused by the size difference of the Ni atoms replacing the Mg in the MgO structure. These distances were chosen considering the pair distances and lattice parameters reported by Kuzmin et al. 1995.[1] The amplitude reduction factor for the Ni atoms was determined to be 0.92 by fitting standard Ni foil EXAFS by fixing the CNs to their

crystallographic values. This value was fixed to determine the CNs in the respective shells of the samples.

Peaks of the Fourier transformed EXAFS data were assigned as 1st: O, 2nd:Mg and Ni, 3rd: O, 4th: Mg and Ni, 5th: O and 6th: Mg and Ni. Peak at ~3 Å has low intensity and therefore could not be assigned unambiguously. In the literature this peak was mentioned and assigned to single scattering paths of O atoms in the 3rd shell and could not be reproduced by ripples of any other scattering paths and therefore assigned to 3rd shell O atoms.

Peaks beyond the 3rd shell consist of many multiple and single scattering paths. After a series of trial and error fits. These peaks were reproduced by using Ni - O1.1 - Mg1.2, Ni - O1.1 - Mg1.2 - O1.1, Ni - O1.3, Ni - O1.1 - O1.3, Ni - O1.1 Mg1.1, Ni - Ni1.3, Ni - Mg1.3 paths for 10% Ni/MgO CP sample, Ni - O1.1 - Ni1.2, Ni - O1.1 - Mg1.2, Ni - O1.1 - Mg1.2 - O1.1, Ni - O1.3, Ni - O1.1 - O1.3, Ni - O1.1 Mg1.1, Ni - Ni1.3, Ni - Mg1.3 for 20% Ni/MgO CP sample, Ni - O1.1 - Mg1.2, Ni - O1.1 - Mg1.2 - O1.1, Ni - O1.3, Ni - O1.1 - O1.3, Ni - O1.1 Mg1.1, Ni - Ni1.3, Ni - Mg1.3 for 10% Ni/MgO WI sample and Ni - O1.1 - Mg1.2, Ni - O1.1 - 01.1, Ni - Mg1.1 - O1.2, Ni - O1.1 - Mg1.2 - O1.1, Ni - O1.3, Ni - O1.1 - O1.3, Ni - Mg1.1 - O1.3, Ni - O1.1 Mg1.1, Ni - Ni1.3, Ni - Mg1.3 20% Ni/MgO WI sample. In these refinements CN of O atoms in the 1st and 3rd shells were freely refined while last shell was fixed to 24 to reduce number of parameters. CN numbers of Ni and Mg atoms were freely refined but their total was fixed to 12, 6 and 24 according to the information obtained from Rietveld and PDF refinements. The CNs of the multiple scattering paths were fixed to their expected values from the FEFF calculations. Fourier transform and fitting ranges were kept constant between samples. Energy shift determination was performed in two steps (1st: Determined as the first peak of the derivative of XAFS data, then refined up to 2nd coordination shell, 2nd: calculated values were modified according to refinement. EXAFS fitting parameters were obtained in the physically

meaningful range: $0.75 < S_0^2 < 1.0$; $\Delta R < 0.1$ Å; $0.003 < \sigma^2 < 0.02$ Å^2 ; $\Delta E_0 < 10$ eV; R factor < 0.02 (Tables S1 and S2).

Section S2. Crystallite size determination via XRD

The Scherrer method was employed to estimate the crystallite sizes of reduced Ni particles and MgO support at 650 ˚C under reaction conditions. The results are significantly different from the ex-situ STEM results that were conducted at room temperature after the reaction. The harsh reduction treatment prior to ammonia decomposition also results in the sintering of the MgO support to 44 nm for 10% Ni/MgO CP in Group 2 (40 nm in Group 3) and 30 nm for 10% Ni/MgO WI in Group 2 (21 nm in Group 3), which does not change further during catalysis. Table S3 contains the volume-averaged crystallite sizes for the Ni particles and MgO support obtained at 650 ˚C during ammonia decomposition. Ni particles in the mild activated samples (Group 1) reach to approximately 10 nm without significant differences between them. Ni particles in the in situ harsh activated samples (group 2) form bigger crystallites for WI samples compared to CP samples. External harsh activation samples (Group 3) show significant differences in both MgO support and Ni particle crystallite sizes, once again proving the importance of in situ studies as the crystallite sizes changes during cooling or exposure to air as the in situ studies have shown.

Supplementary Figures

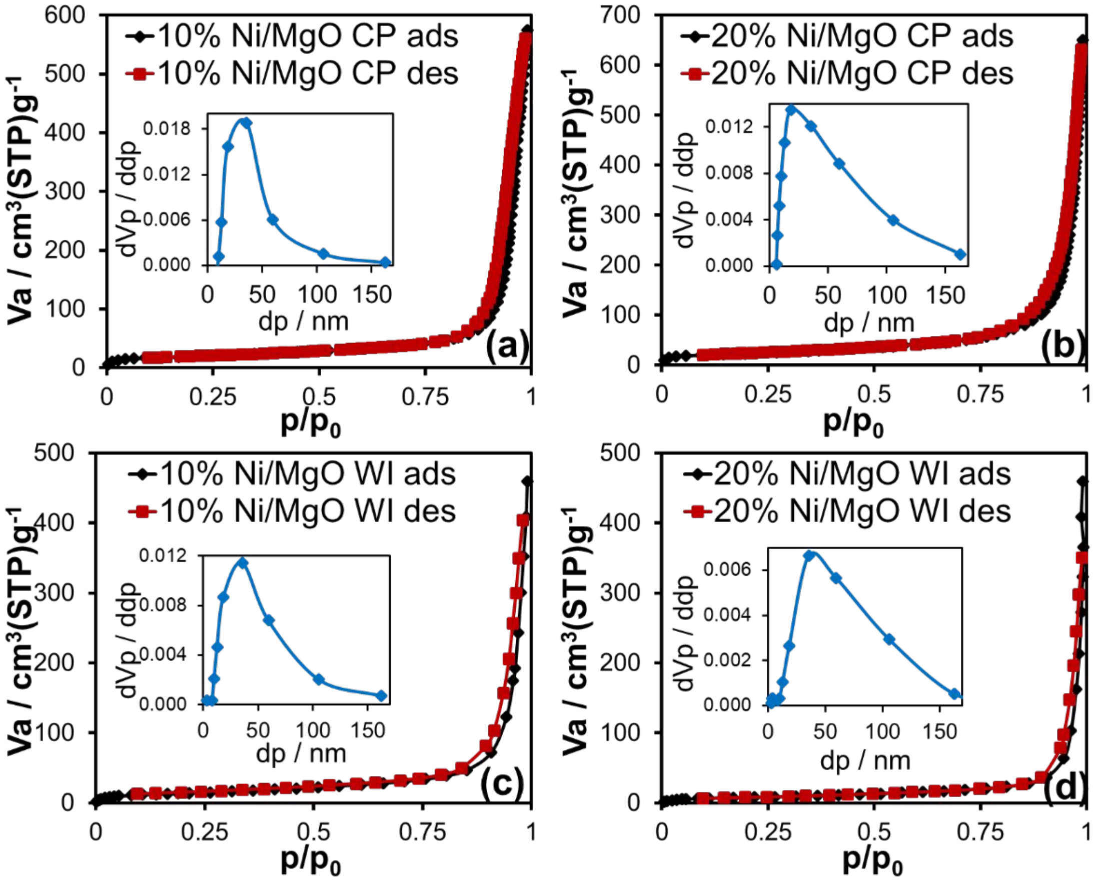

Figure S1. N_2 physisorption isotherms of both co-precipitated (CP) and wet impregnated (WI) Ni/MgO samples with 10at. % and 20at. % Ni loading and their corresponding pore size distributions (insets).

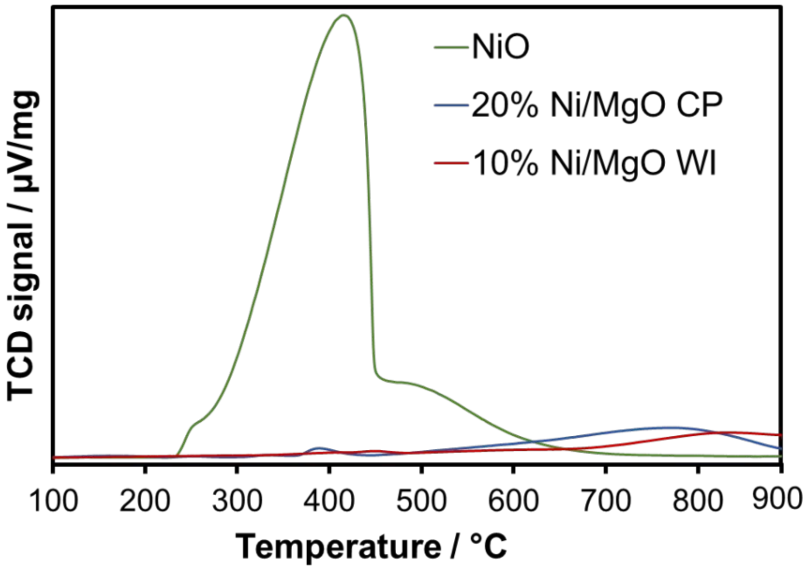

Figure S2. H_2-TPR profiles of NiO, 20% Ni/MgO CP and 10% Ni/MgO WI samples.

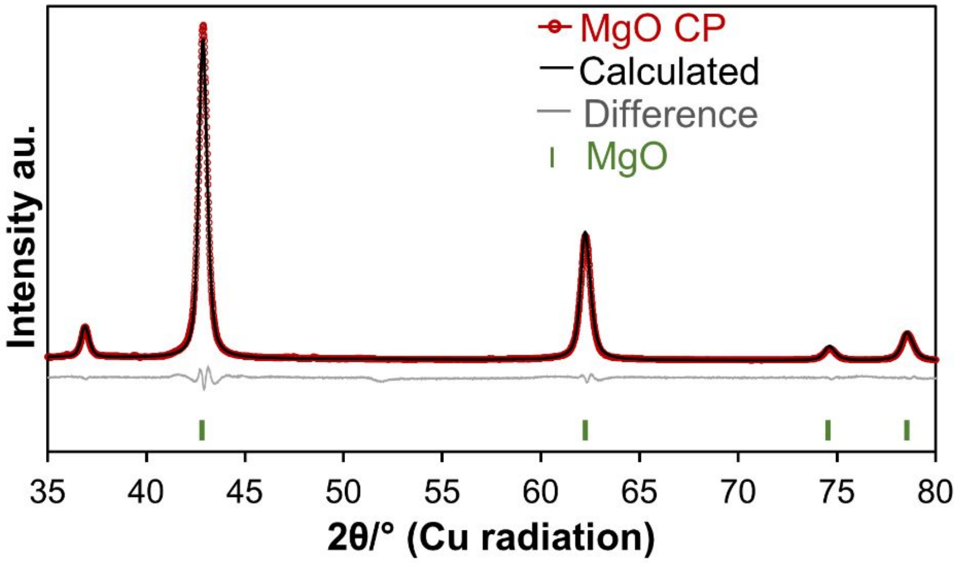

Figure S3. Measured and refined X-ray powder diffraction pattern of MgO synthesized by co-precipitation.

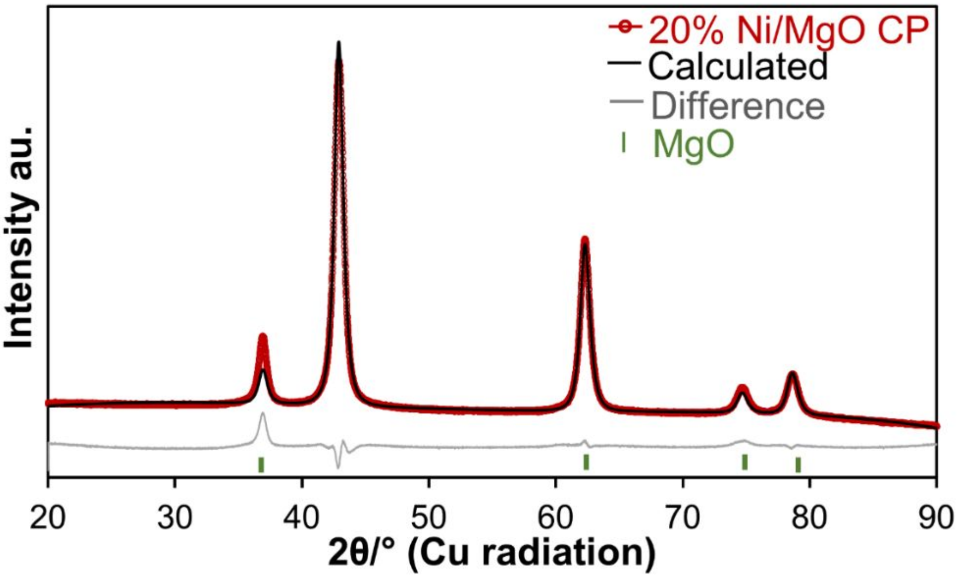

Figure S4. Measured and refined X-ray powder diffraction pattern of the sample 20% Ni/MgO calcined, for Rietveld refinement only MgO was considered as crystalline phase.

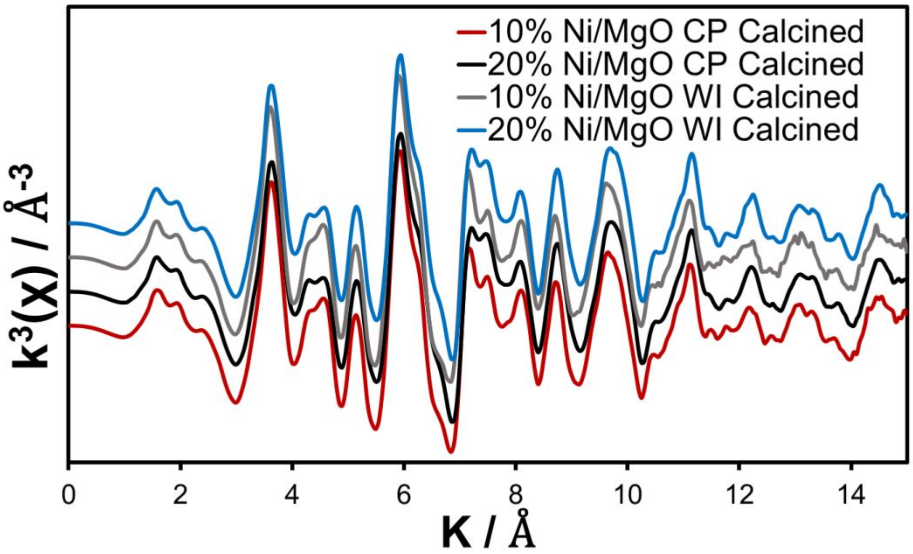

Figure S5. XAFS spectra plotted in k-space for all calcined samples with k weighting of 3.

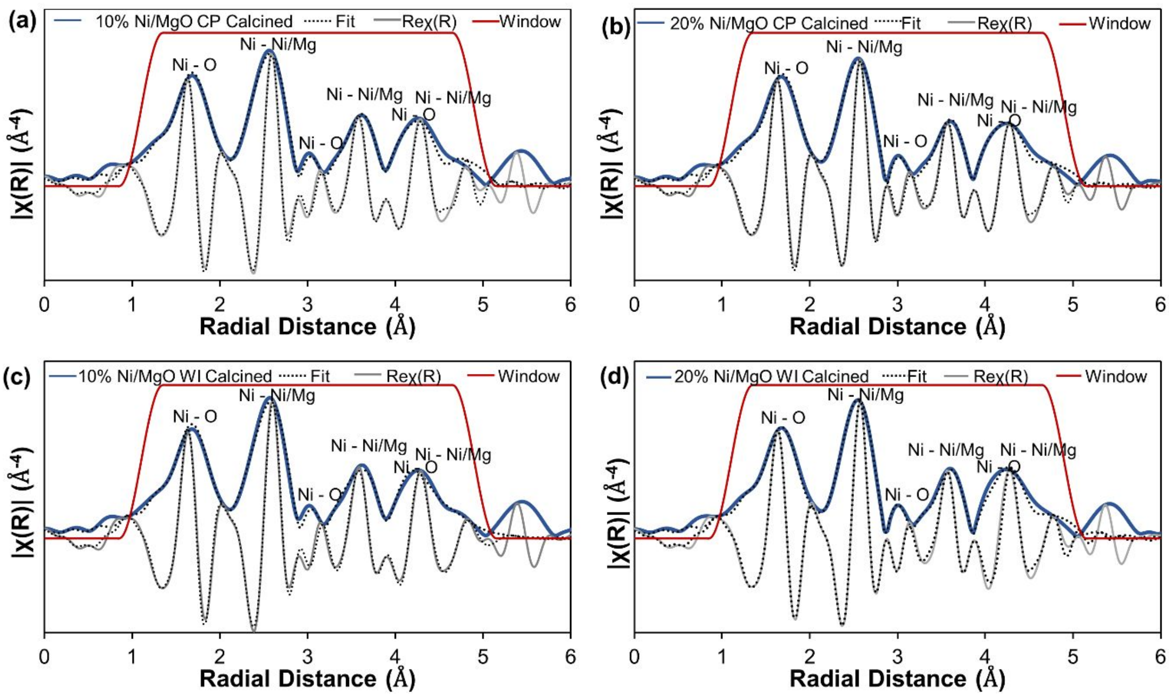

Figure S6. Fourier transform of k^3 weighted Ni K-edge EXAFS of (a) 10%, (b) 20% Ni/MgO CP calcined sample, (c) 10%, (d) 20% Ni/MgO WI calcined sample with EXAFS fit, fitting window, and highlighted atomic interactions. Ni/Mg refers to the total of both Ni-Ni and Ni-Mg interactions.

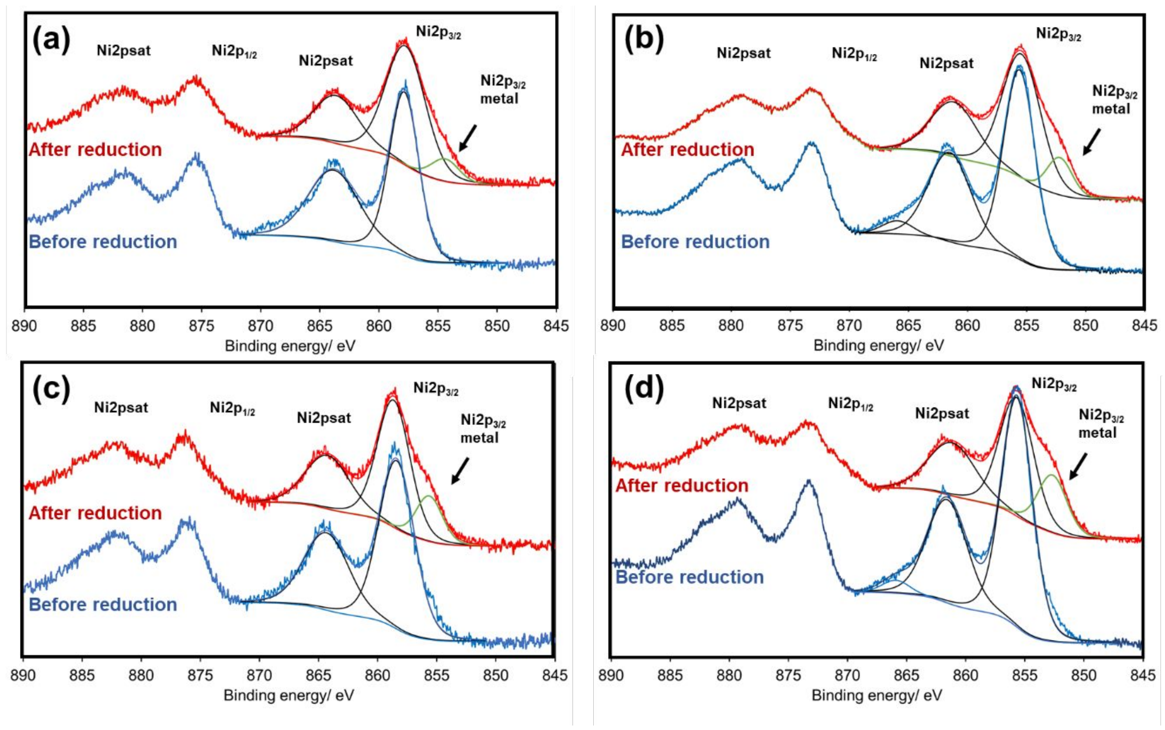

Figure S7. Ni2p core level spectra collected before and after reduction treatment in 10% H2 in N2 at 650 °C for 2 h: (a) CP 10 %, (b) CP 20 %, (c) WI 10 % and WI 20 %.

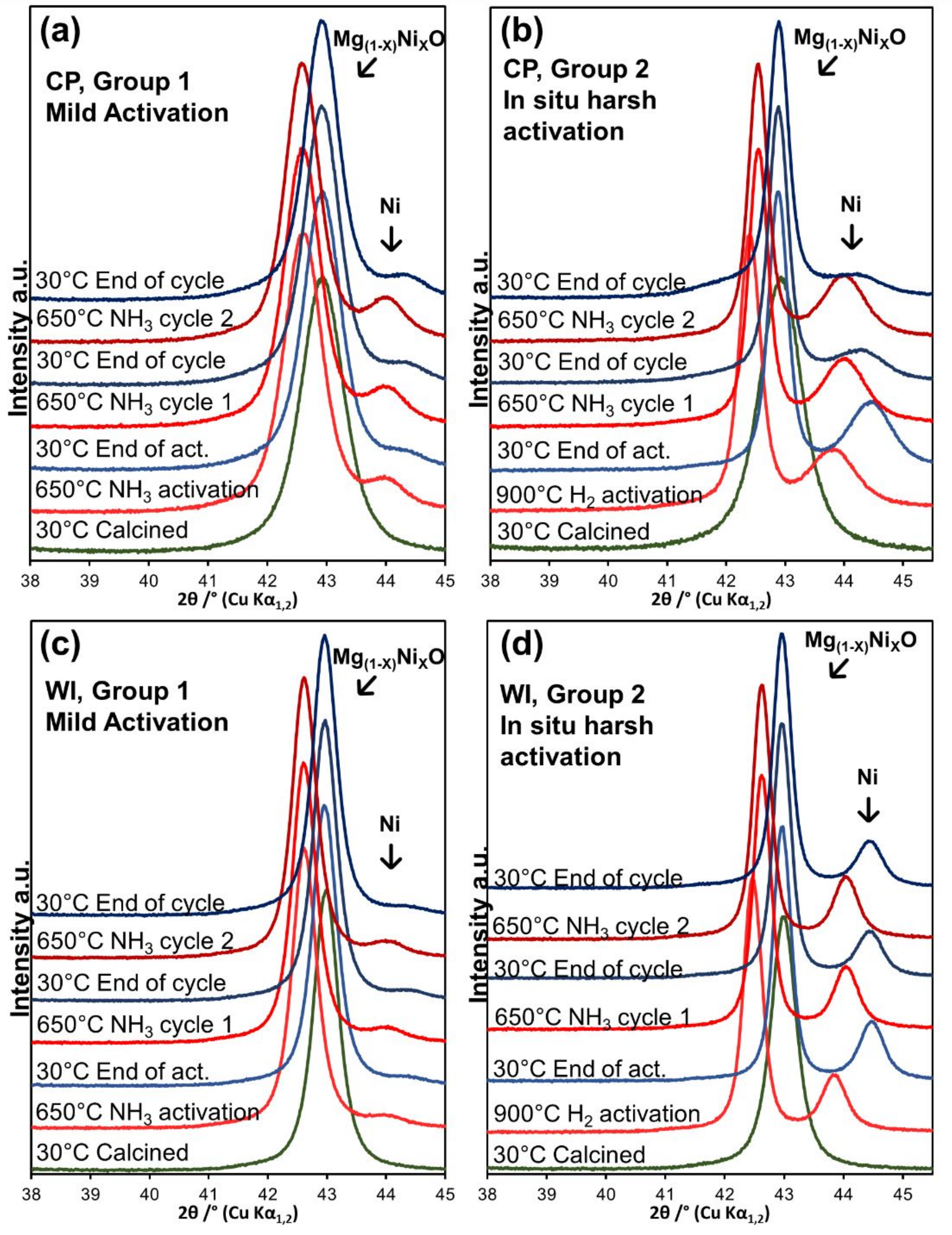

Figure S8. Sections of the in situ XRD powder patterns of 20% Ni/MgO CP activated under (a) mild conditions, (b) harsh conditions and 20% Ni/MgO WI activated under (c) mild conditions and (d) harsh conditions.

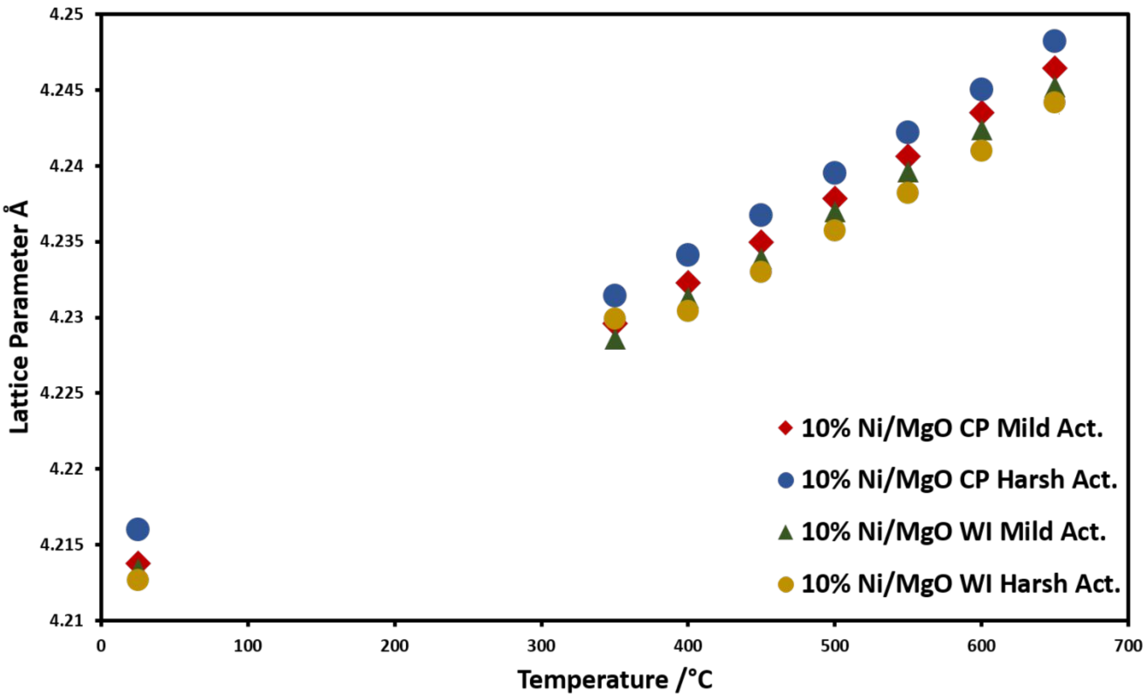

Figure S9. Refined lattice parameters for the MgO phases in 10 % Ni/MgO CP and WI samples activated under mild and harsh conditions.

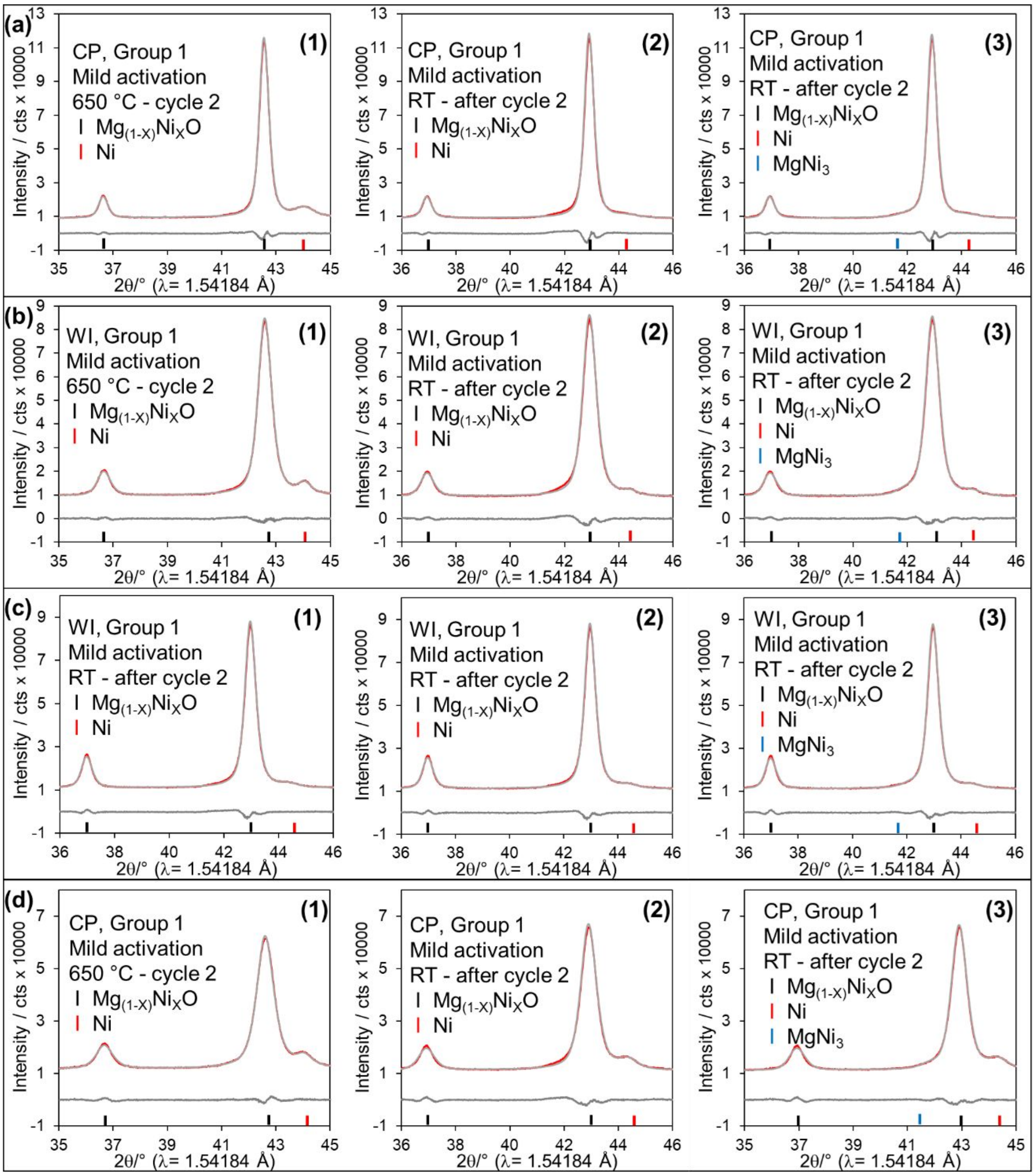

Figure S10. X-ray powder diffraction patterns collected during in situ activation and ammonia decomposition for Group 1 samples. (a) 10% Ni/MgO CP, (b) 10% Ni/MgO WI, (c) 20% Ni/MgO WI, (d) 20% Ni/MgO CP. For each sample, (1) represent data collected at 650 °C at 2nd catalytic cycle, (2) at room temperature after cooling down refined without $MgNi_3$ phase and (3) at room temperature after cooling down refined with $MgNi_3$ phase.

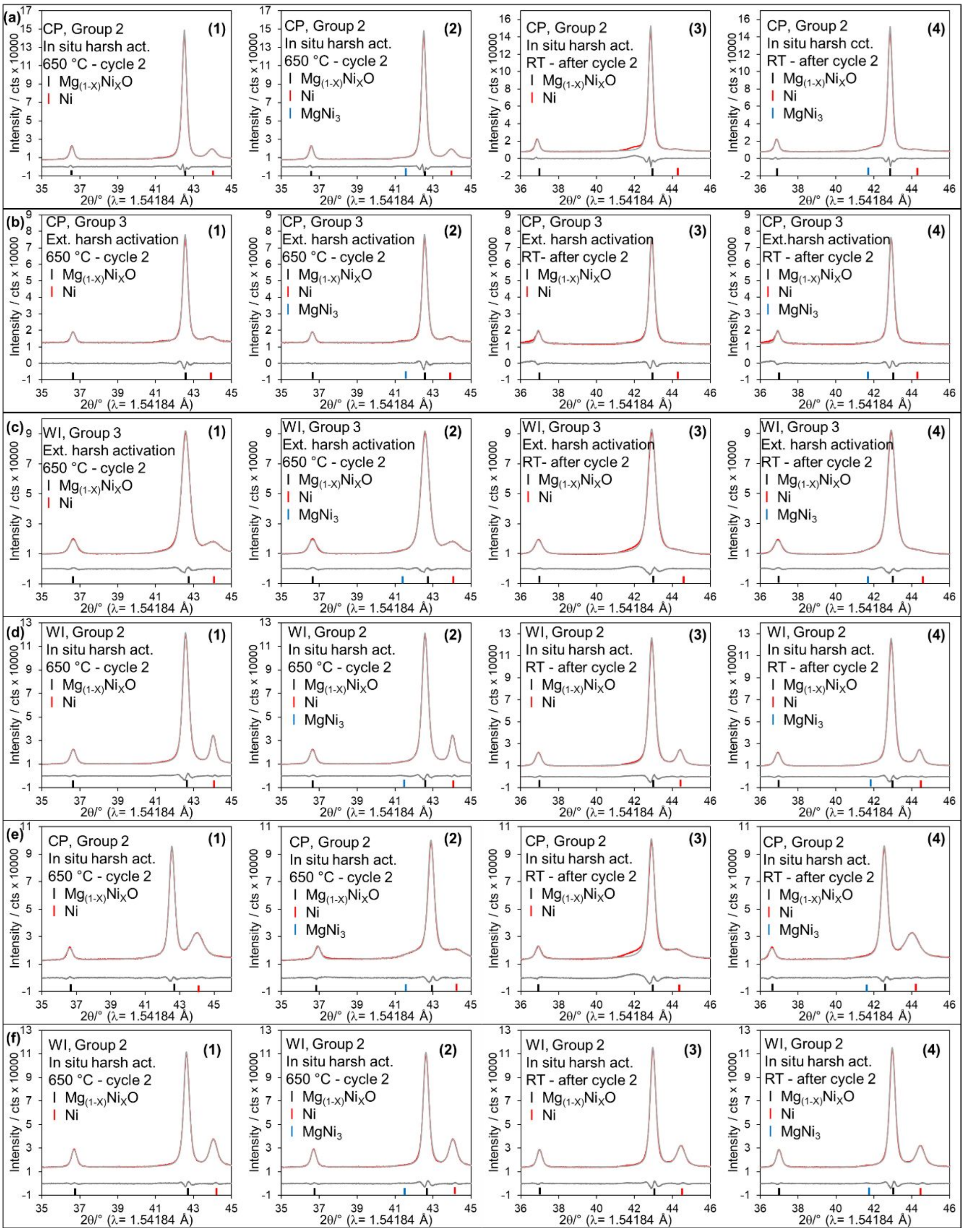

Figure S11. X-ray powder diffraction patterns collected during in situ activation and ammonia decomposition for Group 2 and Group 3 samples. (a) 10% Ni/MgO CP Group 2, (b) 10% Ni/MgO CP Group 3, (c) 10% Ni/MgO WI Group 3, (d) 10% Ni/MgO WI Group 2, (e) 20% Ni/MgO CP Group 2, (f) 20% Ni/MgO WI Group 2. For each sample, (1) represent data collected at 650 °C at 2nd catalytic cycle refined without $MgNi_3$ phase, (2) represent data collected at 650 °C at 2nd catalytic cycle refined with $MgNi_3$ phase, (3) at room temperature after cooling down refined without $MgNi_3$ phase and (4) at room temperature after cooling down refined with$MgNi_3$ phase

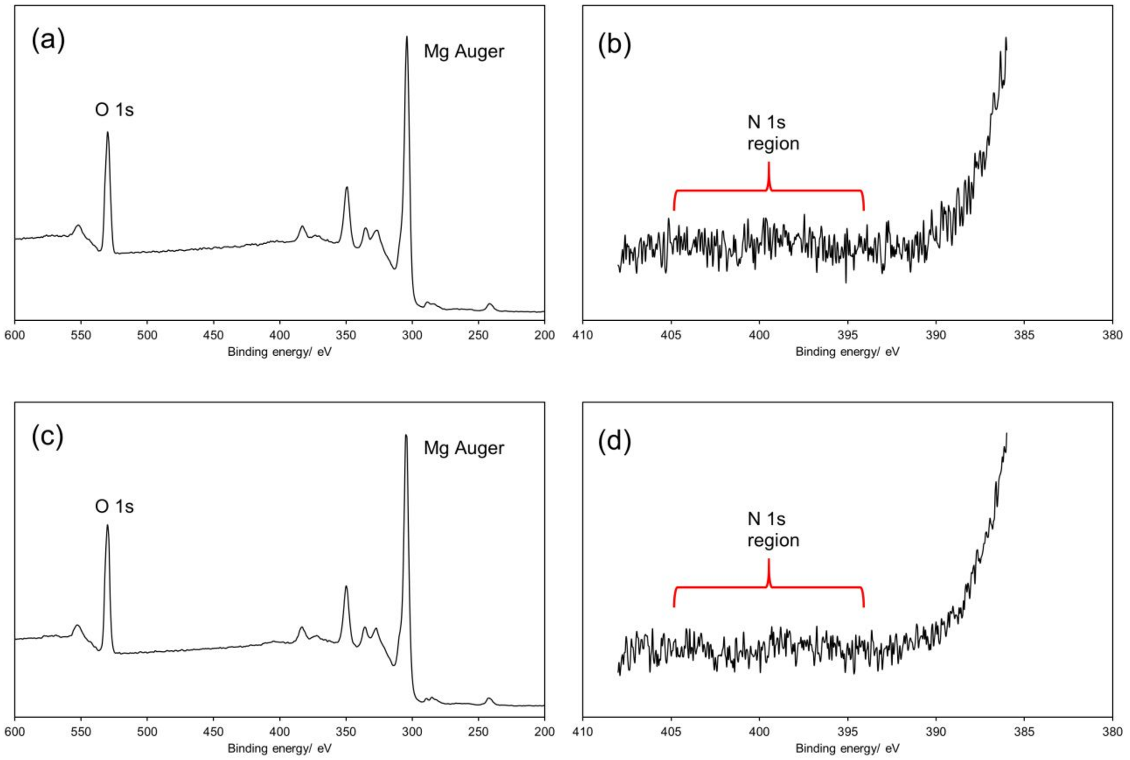

Figure S12. (a) Survey XP scan measured for 10% Ni/MgO CP after harsh activation and two cycles of ammonia decomposition, and (b) high resolution core level spectra N1s. (c) Survey XP scan measured for 20% Ni/MgO CP after harsh activation, and (d) high resolution core level spectra N1s.

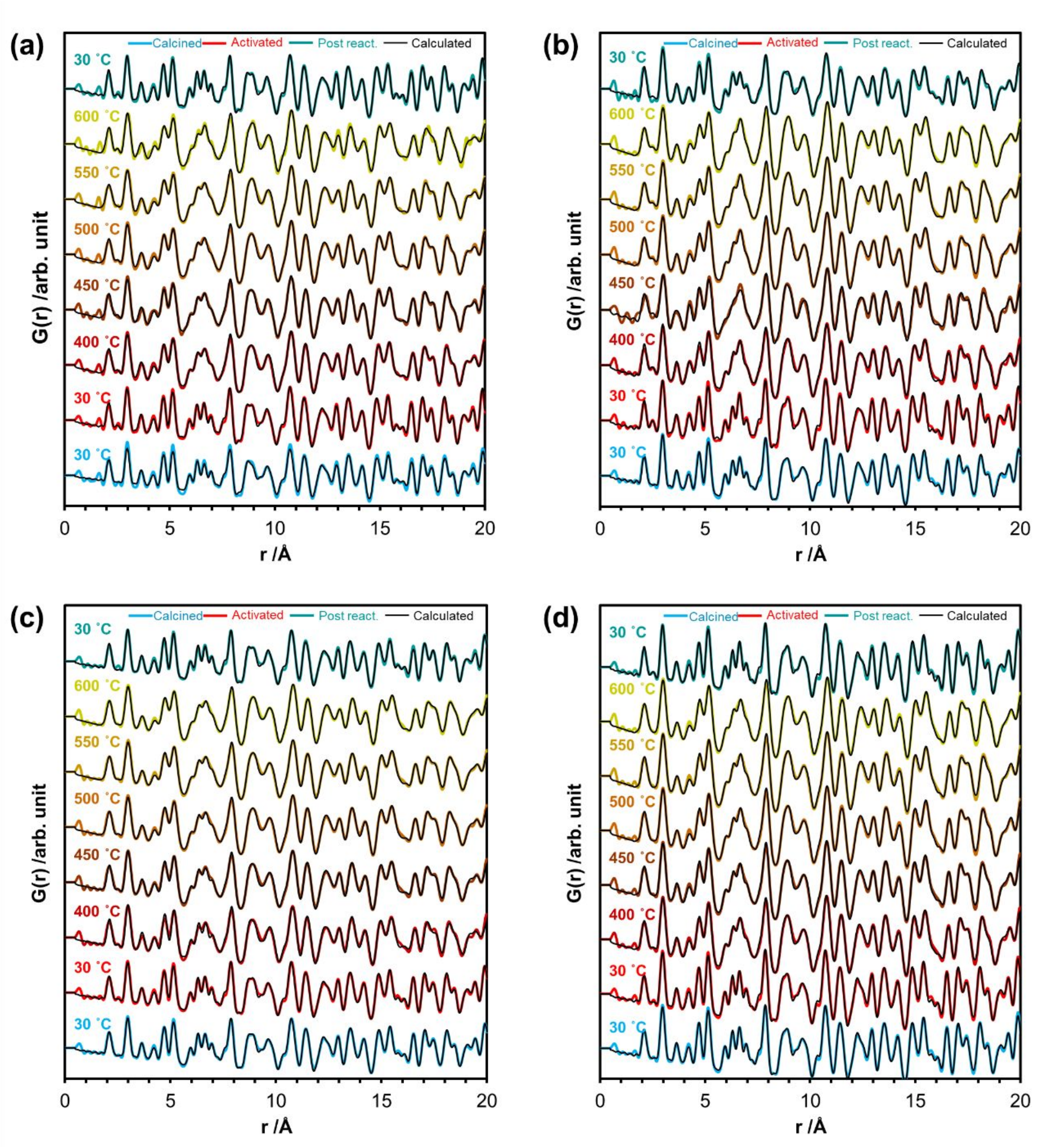

Figure S13. Refined experimental PDFs obtained from samples calcined and given with the results of the PDF refinements: 10% Ni/MgO CP (a), 20% Ni/MgO CP (b), 10% Ni/MgO WI (c) and 20% Ni/MgO WI (d) activated under harsh conditions (Group 3). Note that the bottom-up stacked PDFs correspond to the data collected at 30 ˚C from the calcined and activated samples, at temperatures between 400-600 ˚C in intervals of 50 ˚C as the reaction proceeds, with the data collected at 30 ˚C after cooling down.

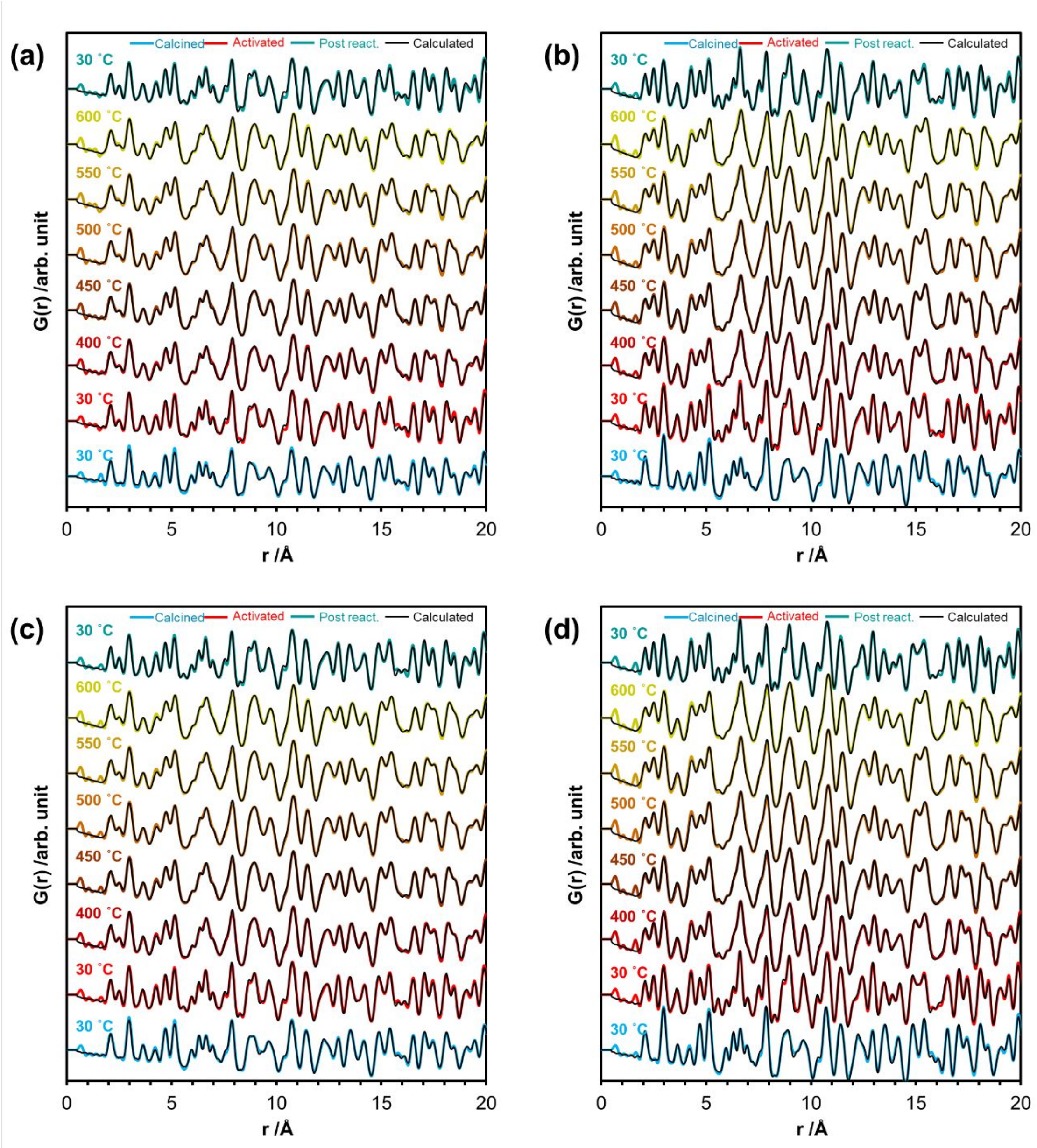

Figure S14. Experimental and calculated PDFs obtained from samples calcined and given with the results of the PDF refinements: 10% Ni/MgO CP (e), 20% Ni/MgO CP (f), 10% Ni/MgO WI (g) and 20% Ni/MgO WI (h) activated under mild conditions (Group 1). Note that the bottom-up stacked PDFs correspond to the data collected at 30 ˚C from the calcined and activated samples, at temperatures between 400-600 ˚C in intervals of 50 ˚C as the reaction proceeds, with the data collected at 30 ˚C after cooling down.

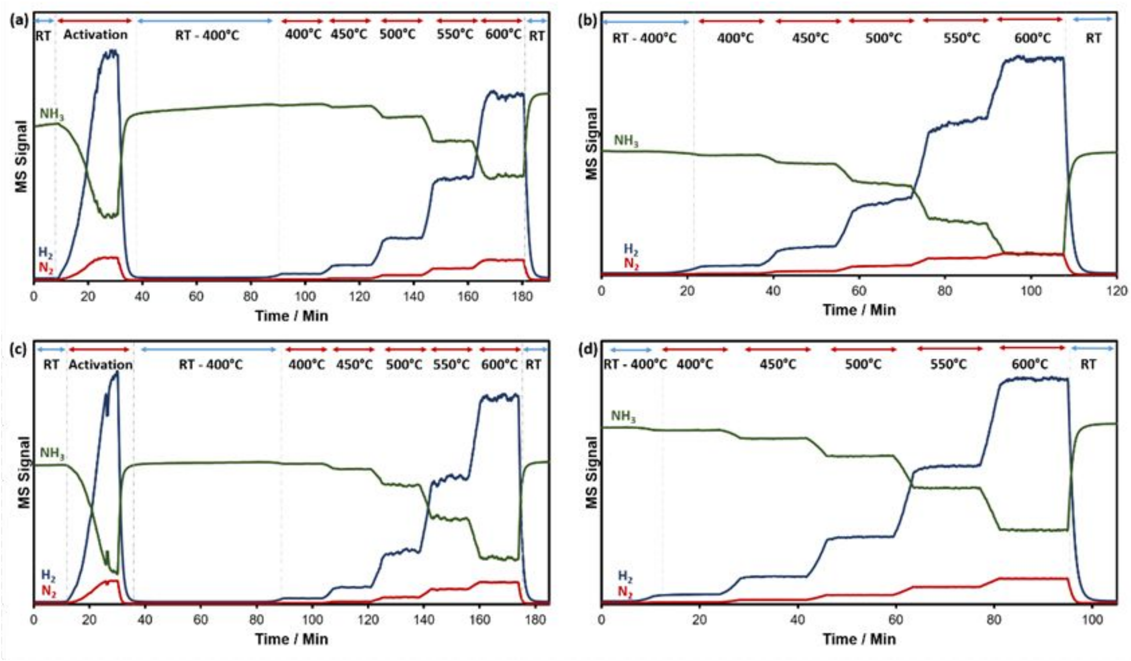

Figure S15. Mass spectrometry (MS) data collected during operando total scattering experiments for 10% Ni/MgO samples. (a) CP samples activated at the beamline under mild conditions, (b) CP samples activated before the measurement externally under harsh conditions. (c) WI samples activated under mild conditions and (d) WI samples activated under harsh conditions.

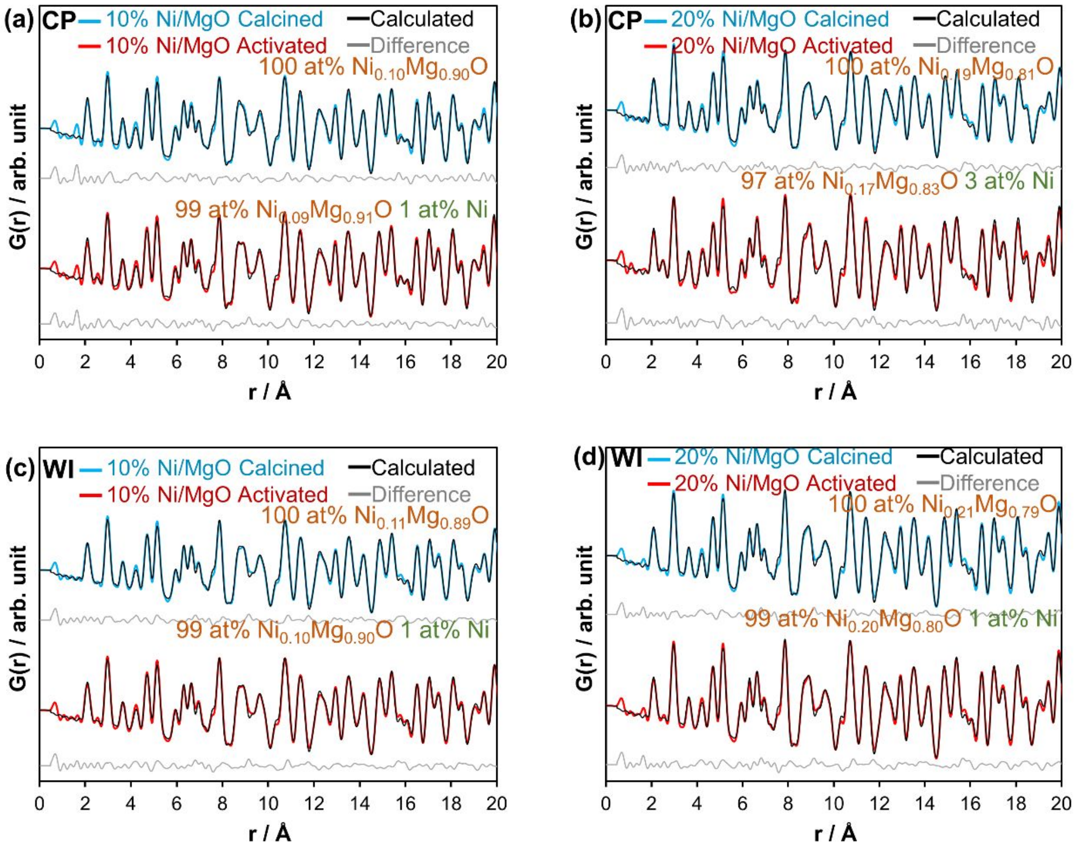

Figure S16. Experimental and calculated PDFs together with their differences, obtained from samples calcined and activated under mild conditions given with the results of the PDF refinements: 10% Ni/MgO CP calcined (a) and activated (b), 20% Ni/MgO CP calcined (c) and activated (d), 10% Ni/MgO WI calcined (e) and activated (f) and 20% Ni/MgO WI calcined (g) and activated (h).

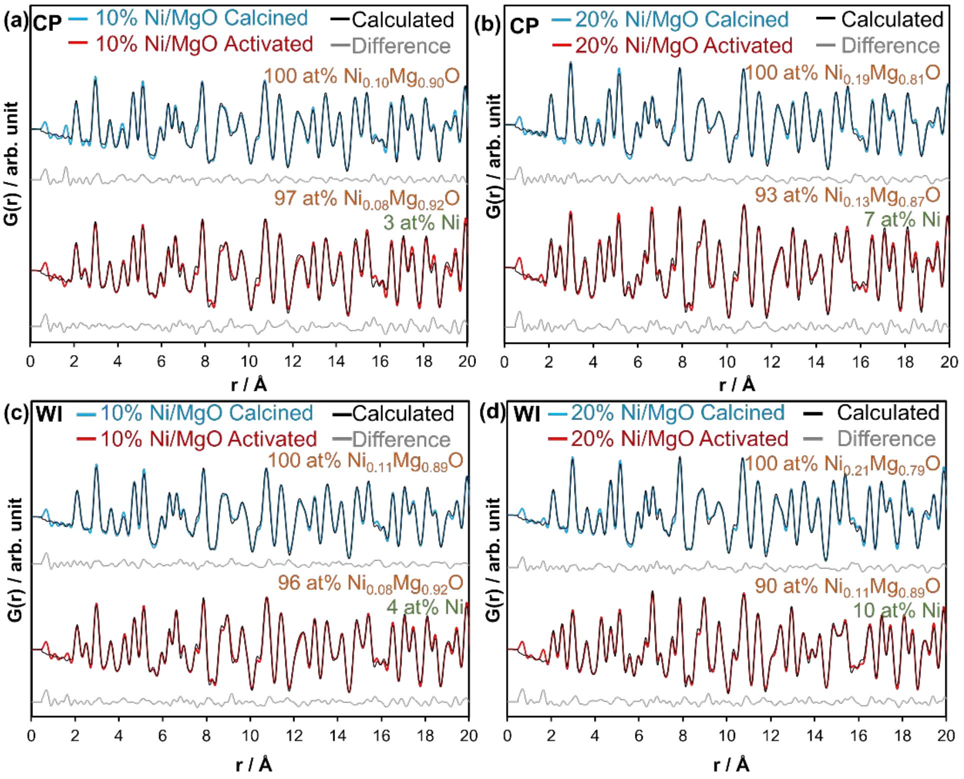

Figure S17. Experimental and calculated PDFs together with their differences, obtained from samples calcined and activated under harsh conditions, given with the results of the PDF refinements: (a) 10% Ni/MgO CP calcined and (b) externally activated, (c) 20% Ni/MgO CP calcined and (d) externally activated, (e) 10% Ni/MgO WI calcined and (f) externally activated and (g) 20% Ni/MgO WI calcined and (h) externally activated.

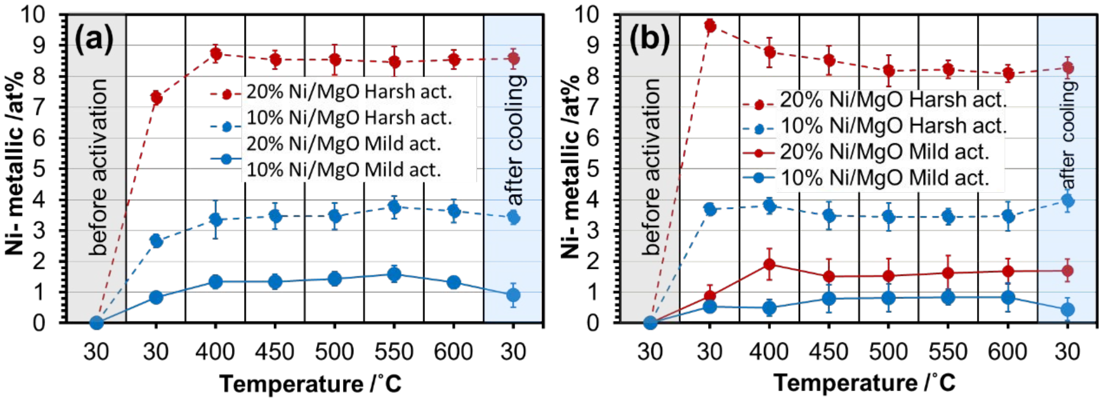

Figure S18. The amount of Ni in at% reduced after harsh and mild activation conditions during the catalytic reaction was calculated from PDF refinements for samples prepared by CP (a) and WI (b).

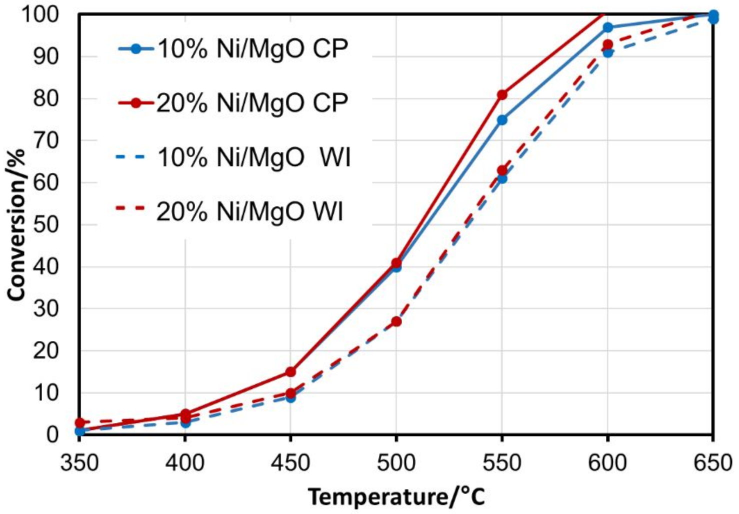

Figure S19. Catalytic test results of co-precipitated and wet impregnated samples activated under 10 % H_2/N_2 flow without exposure to ambient conditions prior to catalytic testing. NH3 conversion curves belong to the second decomposition cycle after activation.

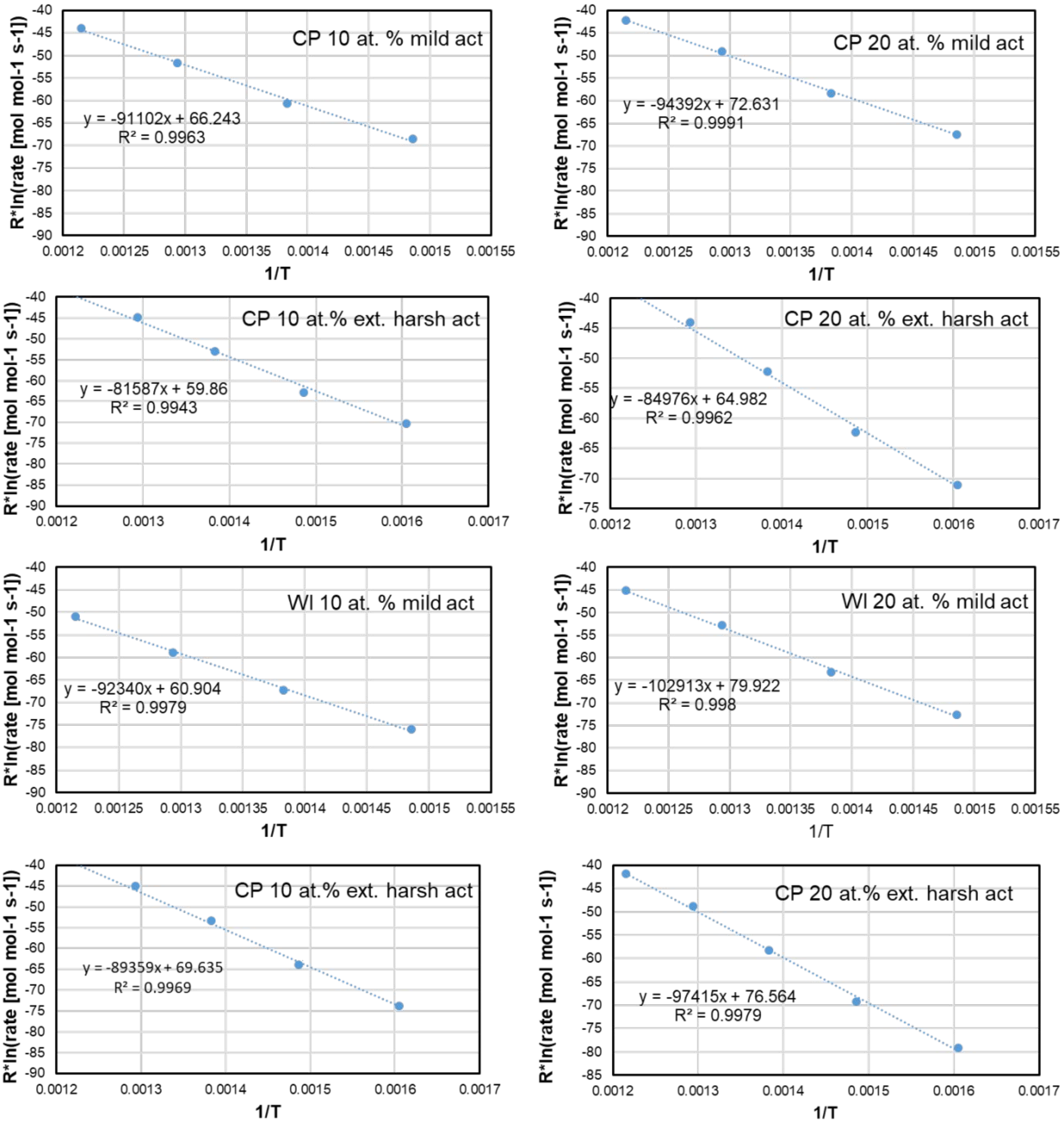

Figure S20. Arrhenius plots for (a) 10% NiMgO CP mild activation, (b) 20% NiMgO CP mild activation, (c) 10% NiMgO CP external harsh activation, (d) 20% NiMgO CP ext. harsh activation, (e) 10% NiMgO WI mild activation, (f) 20% NiMgO WI mild activation, (g) 10% NiMgO WI ext. harsh activation, (a) 20% NiMgO CP ext. harsh activation. Arrhenius plots were drawn in the temperature range of 350 to 550°C.

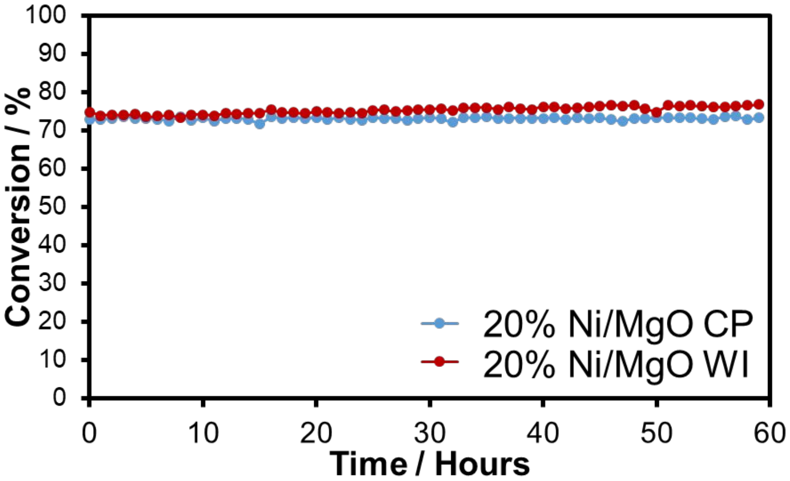

Figure S21. Stability tests for the 20% Ni/MgO samples were studied at around 70 % conversion at 525°C for CP and 565 °C for WI samples and WHSV of 15000 $cm^3\ g_{Ni}^{-1}\ h^{-1}$.

Supplementary Tables

Table S1. EXAFS fitting parameters at Ni K edge for CP samples.

Sample	Scattering path	R (Å)	σ^2 (Å^2•10^{-2})	ΔE_0 (eV)	R factor
10% Ni/MgO CP	Ni - O1.1	2.09±0.01	0.62±0.1	0.3±0.6	0.006
	Ni - Ni1.1	2.96±0.02	0.78±0.1		
	Ni - Mg1.1	2.97±0.03	0.78±0.1		
	Ni - O1.2	3.62±0.03	0.69±0.3		
	Ni - Ni1.2	4.19±0.04	0.33±0.3		
	Ni - Mg1.2	4.21±0.03	0.33±0.3		
	Ni - O1.1 - Mg1.2	4.18±0.03	0.50±0.1		
	Ni - O1.1 - Mg1.2 - O1.1	4.18±0.03	0.5±0.1		
	Ni - O1.3	4.67±0.03	0.78±0.3		
	Ni - O1.1 - O1.3	4.86±0.03	0.35±0.3		
	Ni - O1.1 Mg1.1	4.86±0.03	0.35±0.3		
	Ni - Ni1.3	5.12±0.05	0.98±0.2		
	Ni - Mg1.3	5.16±0.03	0.98±0.2		
20% Ni/MgO CP	Ni - O1.1	2.09±0.01	0.64±0.1	0.7±0.4	0.003
	Ni - Ni1.1	2.95±0.05	0.63±0.1		
	Ni - Mg1.1	2.98±0.01	0.63±0.1		
	Ni - O1.2	3.62±0.03	0.86±0.3		
	Ni - Ni1.2	4.18±0.01	0.23±0.2		
	Ni - Mg1.2	4.19±0.02	0.23±0.2		
	Ni - O1.1 - Ni1.2	4.18±0.01	0.41±0.1		
	Ni - O1.1 - Mg1.2	4.19±0.02	0.41±0.1		
	Ni - O1.1 - Mg1.2 - O1.1	4.19±0.02	0.41±0.1		
	Ni - O1.3	4.68±0.02	0.83±0.1		
	Ni - O1.1 - O1.3	4.86±0.03	0.48±0.3		
	Ni - O1.1 Mg1.1	4.86±0.03	0.48±0.3		
	Ni - Ni1.3	5.12±0.01	0.83±0.1		
	Ni - Mg1.3	5.13±0.03	0.83±0.1		

R: Bond distance for single scattering paths and half of scattering path distance for multiple scattering paths.

σ^2: Debye-Waller Factor

ΔE_0: Energy shift (Inner potential correction)

R factor: Goodness of fit.

Table S2. EXAFS fitting parameters at Ni K edge for WI samples.

Sample	Scattering path	R (Å)	σ^2 (Å^2 •10^{-2})	ΔE_0 (eV)	R factor
10% Ni/MgO WI	Ni - O1.1	2.09±0.01	0.60±0.1	0.1±0.6	0.013
	Ni - Ni1.1	2.93±0.02	0.73±0.1		
	Ni - Mg1.1	2.96±0.02	0.73±0.1		
	Ni - O1.2	3.62±0.03	0.61±0.5		
	Ni - Ni1.2	4.15±0.03	0.84±0.2		
	Ni - Mg1.2	4.18±0.03	0.84±0.2		
	Ni - O1.1 - Mg1.2	4.18±0.03	0.84±0.2		
	Ni - O1.1 - Mg1.2 - O1.1	4.18±0.03	0.84±0.2		
	Ni - O1.3	4.68±0.03	0.77±0.2		
	Ni - O1.1 - O1.3	4.86±0.03	0.20±0.2		
	Ni - O1.1 Mg1.1	4.86±0.03	0.35±0.3		
	Ni - Ni1.3	5.08±0.03	0.35±0.3		
	Ni - Mg1.3	5.12±0.03	0.35±0.3		
20% Ni/MgO WI	Ni - O1.1	2.10±0.01	0.56±0.1	0.5±0.7	0.008
	Ni - Ni1.1	2.93±0.02	0.28±0.2		
	Ni - Mg1.1	2.96±0.01	0.28±0.2		
	Ni - O1.2	3.61±0.03	1.0±0.6		
	Ni - Ni1.2	4.16±0.02	0.30±0.3		
	Ni - Mg1.2	4.19±0.02	0.30±0.3		
	Ni - O1.1 - Mg1.2	4.19±0.02	0.74±0.2		
	Ni - O1.1 - 01.1	4.19±0.02	0.74±0.2		
	Ni - Mg1.1 - O1.2	4.19±0.02	0.74±0.2		
	Ni - O1.1 - Mg1.2 - O1.1	4.19±0.02	0.74±0.2		
	Ni - O1.3	4.69±0.02	0.64±0.2		
	Ni - O1.1 - O1.3	4.87±0.06	0.64±0.1		
	Ni - Mg1.1 - O1.3	4.87±0.06	0.64±0.1		
	Ni - O1.1 Mg1.1	4.87±0.06	0.64±0.1		
	Ni - Ni1.3	5.09±0.02	0.32±0.3		
	Ni - Mg1.3	5.13±0.02	0.32±0.3		

R: Bond distance for single scattering paths and half of scattering path distance for multiple scattering paths.

σ^2: Debye-Waller Factor

ΔE_0: Energy shift (Inner potential correction)

R factor: Goodness of fit.

Table S3. Volume-averaged crystallite sizes determined by the Scherrer method from *in situ* XRD data collected during ammonia decomposition at 650 °C.

Sample	Mild activation (Group 1)		*In situ* harsh activation (Group 2)		External harsh activation (Group 3)	
	MgO / nm	Ni / nm	MgO / nm	Ni / nm	MgO / nm	Ni / nm
10% Ni/MgO CP	27(±1)	11(±1)	44(±1)	22(±1)	40(±1)	10(±1)
20% Ni/MgO CP	15(±1)	11(±1)	29(±1)	13(±1)		
10% Ni/MgO WI	17(±1)	13(±1)	30(±1)	32(±1)	21(±1)	13(±1)
20% Ni/MgO WI	22(±1)	8(±1)	33(±1)	22(±1)		

Table S4. Previously reported conventional oxide-supported Ni catalysts.

Catalyst	Ni Loading wt. %	WHSV $cm^3\ gcat^{-1}\ h^{-1*}$	Temperature °C	Conversion %	References
Ni/La-MgO	20	22000	550	≈ 96	[26]
Ni/Al_2O_3	10	15000	550	≈ 45	[45]
Ni/CaO	10	15000	550	≈ 38	[45]
Ni/CeO_2	10	30000	550	≈ 53	[46]
Ni/CeO_2-BN	10	30000	550	≈ 62	[46]
Ni/Y_2O_3-BN	10	30000	550	≈ 58	[46]
Ni/MgO CP	24.9	15000	550	91	This work
Ni/MgO WI	13.4	15000	550	82	This work

*gcat corresponds to the weight of Ni, not the weight of entire catalyst.
